# The Trigger Factor Chaperone Encapsulates and Stabilizes Partial Folds of Substrate Proteins

**DOI:** 10.1371/journal.pcbi.1004444

**Published:** 2015-10-29

**Authors:** Kushagra Singhal, Jocelyne Vreede, Alireza Mashaghi, Sander J. Tans, Peter G. Bolhuis

**Affiliations:** 1 van ’t Hoff Institute for Molecular Sciences, University of Amsterdam, Amsterdam, The Netherlands; 2 Department of Systems Biophysics, FOM Institute AMOLF, Amsterdam, The Netherlands; Fudan University, CHINA

## Abstract

How chaperones interact with protein chains to assist in their folding is a central open question in biology. Obtaining atomistic insight is challenging in particular, given the transient nature of the chaperone-substrate complexes and the large system sizes. Recent single-molecule experiments have shown that the chaperone Trigger Factor (TF) not only binds unfolded protein chains, but can also guide protein chains to their native state by interacting with partially folded structures. Here, we used all-atom MD simulations to provide atomistic insights into how Trigger Factor achieves this chaperone function. Our results indicate a crucial role for the tips of the finger-like appendages of TF in the early interactions with both unfolded chains and partially folded structures. Unfolded chains are kinetically trapped when bound to TF, which suppresses the formation of transient, non-native end-to-end contacts. Mechanical flexibility allows TF to hold partially folded structures with two tips (in a pinching configuration), and to stabilize them by wrapping around its appendages. This encapsulation mechanism is distinct from that of chaperones such as GroEL, and allows folded structures of diverse size and composition to be protected from aggregation and misfolding interactions. The results suggest that an ATP cycle is not required to enable both encapsulation and liberation.

## Author Summary

Trigger Factor (TF) is an ATP-independent chaperone protein that assists in folding and prevents misfolding. Up to now, it is a general unsolved question how chaperones assist in the folding of protein chains. Experimental methods that can probe at the length and timescales of inter-residue interactions are scarce, while the systems are too large—and the folding process too long—to be studied by computer simulations. To overcome these obstacles, the authors performed molecular dynamics simulations at key moments along the folding pathway, and address the changes in the folding and unfolding dynamics of protein chains while in contact with TF. This study provides the first detailed view on a chaperone-protein complex in different stages of folding and offers an explanation for the ability of TF to guide chains to their native state. Moreover, the results demonstrates the role of TF’s flexibility in interacting with a wide range of client states. Overall, it explains how TF can interact with many types of substrates in various stages of folding, without the need for an ATP cycle to switch between encapsulation and liberation of client proteins.

## Introduction

**Fig 1:**
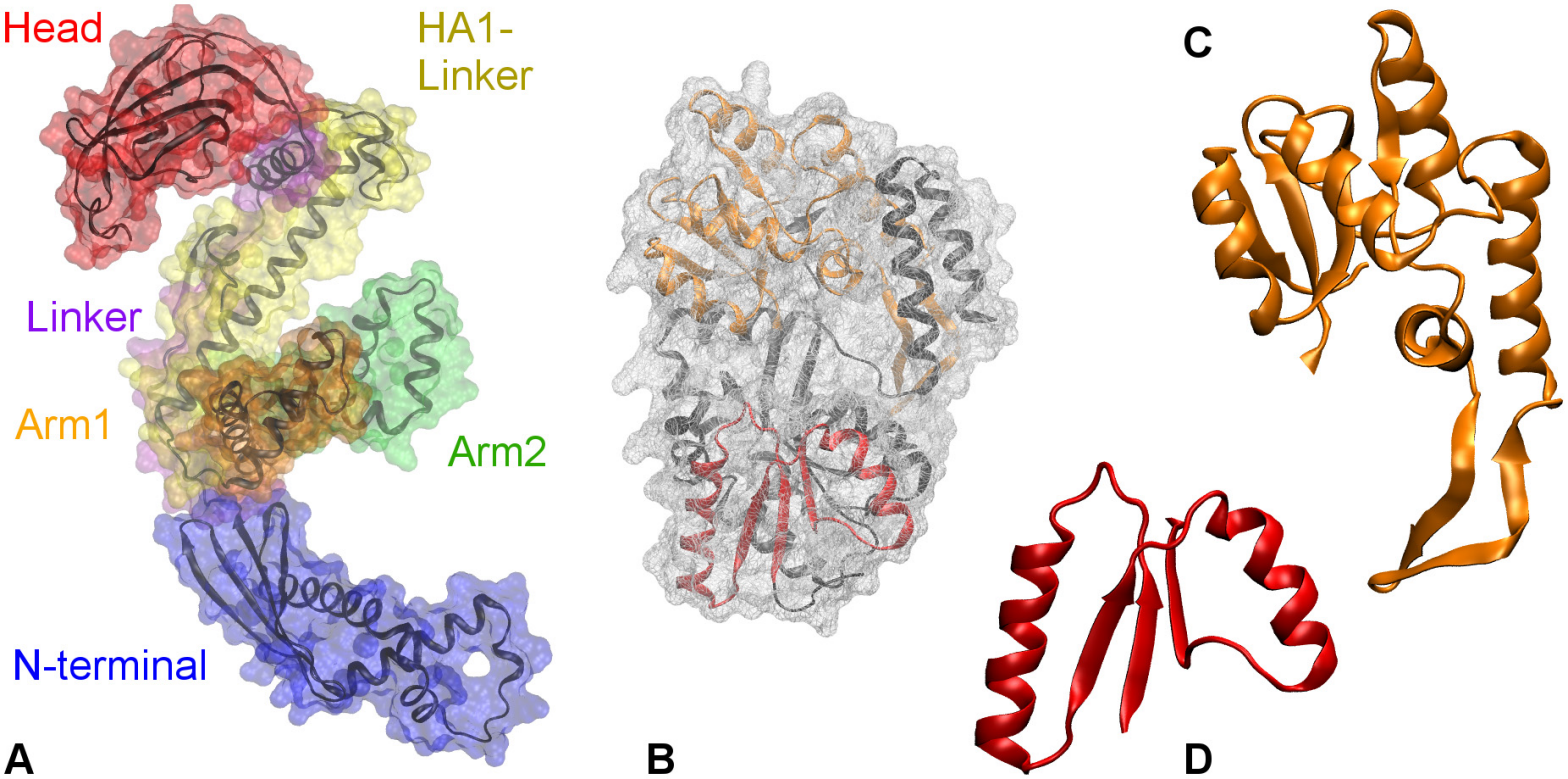
Crystal structures of. **A.** Trigger factor (TF, PDB code: 1W26). The surface of the protein is shown as transparent wire-mesh around the backbone. The surfaces of TF domains are colored differently: N-terminal in blue, Linker in indigo, PPIase domain in red, HA1-linker in yellow, Arm1 in orange, and Arm2 in green; **B.** Maltose binding protein (MBP, PDB code: 1JW4). The surface of MBP is colored gray, while the structure is colored black. Truncate of MBP’s folding intermediates: **C.** P2 with a backbone in orange and **D.** P1 with a backbone in red.

While many proteins can successfully fold independently and spontaneously *in vitro* [1], folding within the cell is facilitated by molecular chaperones [2, 3]. Trigger factor (TF) is a general ATP-independent chaperone [4, 5] found in bacteria (e.g., *E. coli*) [6] and chloroplasts [7, 8]. Bound to the ribosome exit tunnel [9], TF interacts with the emerging nascent chains, and shields them from interactions with other cellular components [10]. TF can also remain associated with polypeptide chains when they are released into the cytosol, and interact with fully folded proteins before their assembly into larger protein complexes [11]. At the ribosome exit tunnel, TF adopts an extended conformation necessary for unfolded substrate interaction [10]. Its dragon-like structure (see Fig 1A) is composed of three domains: N-terminal (residues 1–149); Polypropyl Isomerase or PPIase domain (residues 150–245), also referred to as “Head”; and C-terminal (residues 246–432), which forms the cradle of TF. The C-terminal comprises of a long helical linker (residues 246–302), named “HA1-linker,” with two arm-like extensions—“Arm1” (residues 303–359) and “Arm2” (residues 360–415). A “Linker” (residues 112–149) connects the core of N-terminal (residues 1–111) to the Head. In separate studies, Singhal, *et al*. [12], and Thomas, *et al*. [13], have used molecular dynamics simulations to reveal a surprising structural flexibility enabled by the hinge-like motions of linkers, which can drive a structural collapse of TF in isolation. It has been suggested that its flexibility and promiscuous surface allow TF to interact with substrates of diverse compositions and sizes [8, 11, 14].

How TF interacts with substrates away from the ribosome remains poorly understood. TF is known to bind unfolded and partially folded protein chains [4, 15], and slow down or delay the onset of their folding [16, 17]. Using NMR analysis, Saio *et al*. showed that the sites for TF’s (mostly) hydrophobic interactions with unfolded PhoA are distributed over multiple domains on TF [14]. With optical tweezers experiments [3] on single Maltose Binding Protein (MBP) molecules, Mashaghi *et al*. have shown that that TF not only interacts with unfolded protein chains, but also binds and stabilizes folded structures of different sizes within partially folded proteins [18]. They found that TF binding partially folded structures protects against aggregation interactions, and, hence, promotes folding to the native state. Thus, TF not only acts before the onset of folding, but also during folding as the peptide chain folds step-by-step into a fully folded protein structure. However, it remains unclear how TF binds and stabilizes protein folds at the molecular level, which lies at the basis of its ability to guide the protein chain during folding. Resolving this issue is challenging, given the transient nature of the complexes between TF and partially folded structures.

In this work, we use molecular dynamics (MD) simulations to characterize the interactions and the structural dynamics of the TF-MBP complex. We study the interactions with TF in three distinct stages of MBP’s folding process: fully unfolded protein chains, partially folded states, and native MBP. Fig 1B, 1C and 1D show the structural representations of native MBP and its partially folded states that were employed in this work. All-atom MD simulations of chaperone-assisted protein folding are computationally very expensive due to the large system size and the long time scales connected to relaxation. Using coarse-grained forcefields (1 bead per amino acid), O’Brien *et al*. [19] investigated protein folding at the ribosome, while extending the nascent chain. They showed that TF facilitates folding by decreasing the rate of structural rearrangements within the nascent chain, and increasing the effective exit tunnel length. While greatly extending time scales, this study missed the atomistic details of the interactions of TF with the substrate protein, as well as changes within the substrate proteins. The experimentally observed interactions between TF and partially folded states [18] provided an opportunity to study the mechanisms of interactions between TF and its substrate during the folding process. Therefore, we performed all atom MD simulations on MBP truncates that correspond to the experimentally observed partially folded states—namely, P1 (residues 7–62) and P2 (residues 112–248) [18, 20] (see Fig 1C and 1D). These partially folded truncates owing to their transient stability, provide good model substrates at lower computational cost. In this study, we focus primarily on the dynamics of the smallest partial fold—P1. In addition, we employ steered molecular dynamics simulations on folded P1 to understand the stabilization effect of TF by comparing the potentials of mean force of P1’s unfolding in presence and absence of TF.

We found that TF interacts with folded structures with the ends of its flexible appendages, especially N-terminal, thus forming a “Touching complex”. TF can also leverage its flexibility to wrap its appendages around the folded structures (P1), forming the more stable “Hugging complexes,” while other MBP segments remain unfolded. This binding is the strongest with unfolded protein chains, and weakens gradually with the progressive folding of the polypeptide. It weakens significantly upon the formation of fully folded MBP, which likely plays a role in the post-folding release of the protein. Our steered MD simulations showed that breaking the end-to-end *β*–sheet hydrogen bonds of P1 in these complexes requires more work than when P1 is in isolation. This suggests that by wrapping around partially folded structures and establishing multiple favorable contacts, TF stabilizes these partial folds. In independent MD studies, Singhal, *et al*. [12], and Thomas, *et al*. [13] found that the Arm1 plays an important role in TF’s collapse by strongly interacting with the PPIase domain. Consequently, Thomas, *et al*. speculated that the TF’s collapse may adversely affect its interactions with unfolded chains. Our results show that the binding of unfolded polypeptides is indeed much weaker with the collapsed conformations of TF than with the extended conformation, presumably due to the lack of Arm1’s availability in the former. This highlights the importance of Arm1 in TF’s interactions with unfolded substrate proteins.

While our aim is to provide a molecular view on TF’s interactions during progressively more structured stages of protein folding, the partial folds in our simulation might also be seen as individual proteins. A comparison between the different systems then shows that the N-terminal domain plays an important role in the early interaction stages for all proteins, whereas the precise interaction maps depend on the specific details. Interactions with less structured and more hydrophobic substrates are clearly stronger and more stable.

Our results provide the first view of the atomistic underpinnings of how a chaperone is able to interact with a protein chain during folding and to guide it to its native state. Taken together, they reveal a model of a highly adaptive chaperone, in which TF grabs the partial folds with its domain tips as they form, transfers them to its cradle to encapsulate them so that they are protected against misfolding interactions. TF, thus, acts as a “cradle for partial folds” that uses its flexibility to adjust its structure to the protein substrates of diverse size and composition, and protects the non-folded and partially folded chains against misfolding interactions with other domains or other proteins. It is worth noting that this encapsulation mechanism is distinct from that of chaperones that require ATP for their functioning, such as GroEL/GroES. TF does not require an ATP cycle to enable both substrate encapsulation and liberation in chaperones, and employs its own structural flexibility to achieve the same. This model provides a structural explanation for the ability of TF to interact with and stabilize a wide spectrum of substrates, ranging from unfolded chains to the diverse intermediate folded structures *en route* to the native state [18].

## Methods

### Molecular Dynamics Simulations

Table 1 lists the various systems that were simulated. The systems involve the native structures of full Maltose Binding Protein (MBP) (PDB code: 1JW4), as well as two of its folding intermediates or partial folds—named P1 (residues 7–62) and P2 (residues 113–248). Their structures were acquired from the PDB file for full MBP by removing the other residues.

**Table 1:** MD systems summary.

**TF**	**Substrate**	**#traj**	**#atoms**	**time (ns)**
	*Folded P1*	4	11356	200
	*Unfolded P1*	4	271397	300
	*P2*	4	33077	200
	*MBP*	4	38653	200
Extended	*Unfolded P1*	8	485567	300
Semi-collapsed	*Unfolded P1*	4	312277	300
Fully-collapsed	*Unfolded P1*	4	321958	300
Extended	*Folded P1*	4	262967	300
Semi-collapsed	*Folded P1*	4	235310	300
Fully-collapsed	*Folded P1*	6	235300	300
Docked	*Folded P1*	3	310489	100
Extended	*P2*	6	307981	200
Extended	*MBP*	4	308184	200

Summary of all the AA-MD systems simulated in this work. The Docked Folded P1 set of simulations was performed with the Amber99SB-ILDN forcefield.

We employed Gromacs 4.5 (or 4.6 in some cases) [21] to perform MD simulations of these protein substrates in isolation, as well as with the trigger factor (PDB code: 1W26) in several different starting configurations. The atomic interactions were defined by the Amber03 [22] force field for most systems. In a small set of recently concluded simulations, we used the Amber99SB-ILDN [23] force field in light of the findings of Beuchamp, *et al*. [24]. To all PDB structures, hydrogen atoms were added for the protonation state at pH 7.0. After a steepest descent energy-minimization we solvated the structures in a dodecahedron periodic box (of diameter 15–16 nm) with TIP/3P water molecules for the Amber03 systems, and in TIP/4P water molecules for the Amber99SB-ILDN structures. The box sizes were set to allow for sufficient translational and rotational dynamics of both molecules. The total systems size varied from 11356 atoms for the folded P1 only to almost 500000 atoms for the extended TF with an unfolded P1. The systems were neutralized by adding Na^+^ and Cl^−^ ions (50 mM NaCl). Energy-minimization, followed by 50–100 ps position-restrained (all heavy atoms of protein restrained with force constant of 1000 N/m in each direction) MD runs resulted in equilibrated positions of water molecules and ions. The systems were coupled with a v-rescale thermostat (*τ* = 0.2) and a Parrinello-Rahman barostat (*τ* = 1.0 and reference pressure = 1 Bar). The bonds in the protein were constrained using the LINCS algorithm [25]. Coulombic and van der Waals interactions were treated with a cut-off radius of 1.0 nm, while long-range electrostatic interactions were handled using the PME algorithm (with a mesh size of 0.12 nm). Parallel production runs were performed (with different random starting velocities) for 200–300 ns at close to room temperature (300 K) with a time step of 2.0 fs (coordinates were written every 2.0 ps). Each 200–300 ns long MD simulation trajectory (started with varying random velocities) took on the order 50000 CPU hours.

Four runs were performed on a TF-MBP system, in which the MBP’s crystal structure was placed at a center of mass distance of 8 nm (with minimum distance of approx. 3 nm) from the extended structure of TF. For the set of six runs on TF-P2 complex, the P2 was placed at a center of mass distance of 7 nm (with minimum distance of nearly 2.5 nm) from the extended TF. P1, with 56 residues the smallest of the five partial folds, was studied using several initial conditions of both P1 and TF: three sets of runs for the folded P1 with different starting configurations of TF, and three sets of runs for an unfolded P1 chain with different initial configurations for TF. In the former set of simulations, P1 was placed at a minimum distance of 2.3–3.5 nm from: the extended TF structure (PDB structure), the semi-collapsed (SC-TF) conformation, and the fully-collapsed (FC-TF) conformation (last two obtained from isolated TF simulations [12]). The P1 complex with extended TF was named “Touching Complex.” Similarly, the three sets performed on TF-unfolded P1 systems were also initiated with the unfolded P1 at a minimum distance of 3–4 nm from the three conformations of TF—extended, semi-collapsed, and fully-collapsed. In all TF-P1 systems, four to eight simulations were run for 300 ns, resulting in the total aggregate time of about 15 *μ*s.

The first 200–300 ns provide an overview of the early interactions between TF and its substrates. However, to further capture the post-folding dynamics of P1’s interactions with TF, we bypassed the spontaneous but very slow transition towards complete binding of the substrate to TF. To this end, we used an energy minimized configuration of the TF–P1 complex as an initial conformation to perform additional MD simulations, and acquire insight in the more strongly bound states. To provide such a minimum energy conformation, we performed molecular docking calculations on the TF–P1 complex. Docking calculations with the Rosetta Docking server [26–28] gave 10 minimum energy configurations. Starting from the three lowest energy configurations, we performed three additional 100 ns MD simulations to evaluate the stability of these complexes, called “Hugging Complexes”. Two of the final conformations that we obtained were used as starting structures for pulling experiments.

### Steered MD

We performed steered MD (SMD) calculations on P1 in isolation and two TF-P1 systems by pulling at the ends of the P1 (residues 7 and 62, respectively). PLUMED 2.0 [29] was employed in combination with GROMACS 4.6 and the Amber99SB-ILDN force field [23]. The systems were placed in a dodecahedral water box of 15 nm (and 50mM NaCl) and output frequency of 100 time steps (with time step of 2.0 fs). We attached dummy beads at the two ends through a harmonic spring (with a spring constant of 50 kN/nm), and pulled them apart at a speed of 0.5 nm/ns, unfolding P1 in about 30 ns. To prevent jumps in the pulling coordinate when the distance to a periodic image would be shorter, interactions between periodic images were switched off for the pulling coordinate (although, for all other interactions, periodic boundary conditions are taken into account).

Initial conditions for the SMD runs were obtained from 100 ns AA-MD simulations on the lowest energy Rosetta docked TF-P1 complexes, HC1 and HC2 respectively. All systems were prepared/equilibrated as described above. For each system, we ran 20 steered MD simulations, and obtained the work-extension curves. We computed the average work required for unfolding P1 in each system (〈*W*_0−>*t*_〉), and by applying Jarzynski equality [30], the potential of mean force (PMF) from the exponential average work (*k*_*B*_ *T* log〈exp(−*βW*_0−>*t*_〉). As the PMF is dominated by small values of *W*_0−>*t*_ [31] a better approach is to use the stiff-spring approximation and compute the potential of mean force (PMF) from the first and second cumulant (〈*W*〉 − 0.5*β*(〈*W* ^2^〉−〈*W*〉^2^)) [31]. Note that the second cumulant in the PMF expression is the estimation of variance in work over the extension. Large fluctuations in the work-extension curves, thus, result in spuriously large negative values of PMF, rendering the PMF unreliable.

### Analysis

To analyze the interactions between TF and substrate, we define the formation of a contact when the minimum distance between the heavy atoms of the two molecules falls below 0.4 nm. Such an event is labeled an *attachment* event. In contrast, a *detachment* event was recorded when, after the formation of a contact, the corresponding minimum distance increased above 1.0 nm. The margin prevents registration of false detachment events. The *mean first binding time* of the TF-substrate interaction measures the time, averaged over the number of trajectories, it takes to form a metastable complex after the first contact, i.e., the last attachment event that is not followed by detachment events in rest of the MD simulations.

The probability of a domain or residue of TF to form a contact with substrates (the contact probability) is estimated by determining the area of the normalized probability distribution of the minimum distance between the corresponding heavy atoms on TF and the substrate for values less than 0.4 nm. The number of partial fold contacts, or PF-contacts, in P1 are defined as the number of backbone hydrogen bonds between the *β*-strands formed by first five (residues 7–11) and last five residues (residues 58–62) of P1.

The fraction of hydrophilic contacts was calculated by dividing the number of contacts between nitrogen and oxygen atoms of molecules by total number of contacts between the respective molecules. Hydrophobic maps of molecules were calculated by the novel hydrophobic probes method developed by Acharya *et al*., [32] as described in an earlier work [12]. The hydrophobicity map shows the location and the strength of the hydrophobic patches (or residues) on the TF’s surface.

All analysis was performed using indigenous scripts and the built-in tools of GROMACS 4.5 and 4.6 [21]. VMD 1.9 was used for visualization of protein structures and creating images [33].

### Principal Component Analysis

We concatenated the trajectories of the dynamics of unfolded P1 in isolation and in complex with extended TF, and performed principal component analysis on the dihedral angles of P1 [34] using GROMACS tools. After sorting the common eigenvectors in the descending order of the associated eigenvalues, we projected the concatenated trajectory along the first 15 eigenvectors. The 2-D projection along any pair of eigenvectors was obtained by the plotting the corresponding projections against one another. In this projection, the two sets of simulations (P1 in isolation and P1 in complex with extended TF) can be easily separated to differentiate the conformational spaces explored by them along common eigenvectors. Results of this analysis are discussed in the Supporting Information.

## Results and Discussion

### TF and Unfolded P1

We performed multiple MD simulations of unfolded P1 with TF, which is initially either in extended (crystal structure), semi-collapsed or fully collapsed conformation (see Methods) [12]. The unfolded chain attaches to the TF in almost all MD simulations, though the binding is initially transient, with multiple attachment-detachment events per binding site (see S1 and S2 Figs). The number of these events, and the mean first binding time—defined as the average time after which no detachment events are observed (see Methods)—are the smallest for the unfolded P1-extended TF system (Table 2). The two form a stable complex in a very short time, after detaching only once. These values increase significantly for the semi- and fully-collapsed conformations of TF. This increase can be easily understood, as in the latter two conformations, Arm1 forms a contact with PPIase domain (with an additional interface between N-terminal and Arm2 for fully-collapsed TF), hence can no longer bind to the substrate. In that case, while TF can still bind the substrate proteins, the interactions may be diminished. This is in agreement with Thomas *et al*. [13] who, building up on the work of Gupta *et al*. [35], proposed that the formation of an interface between PPIase domain and Arm1 in isolated TF might affect the interactions between TF and nascent chains.

**Table 2:** Metastability of TF-substrate binding.

**TF**	**Substrate**	*τ*	*N*
*Extended*	*Unfolded P1*	2.75	1
*Semi-Collapsed*	*Unfolded P1*	63.12	10
*Fully-Collapsed*	*Unfolded P1*	58.18	8
*Extended*	*Folded P1*	88.27	12
*Semi-Collapsed*	*Folded P1*	45.03	4
*Fully-Collapsed*	*Folded P1*	27.70	3
*Extended*	*P2*	97.03	14
*Extended*	*MBP*	158.03	76

For each TF-substrate system: Mean First Binding Time (*τ*, ns); Number of attachment-detachment events (N).

**Fig 2:**
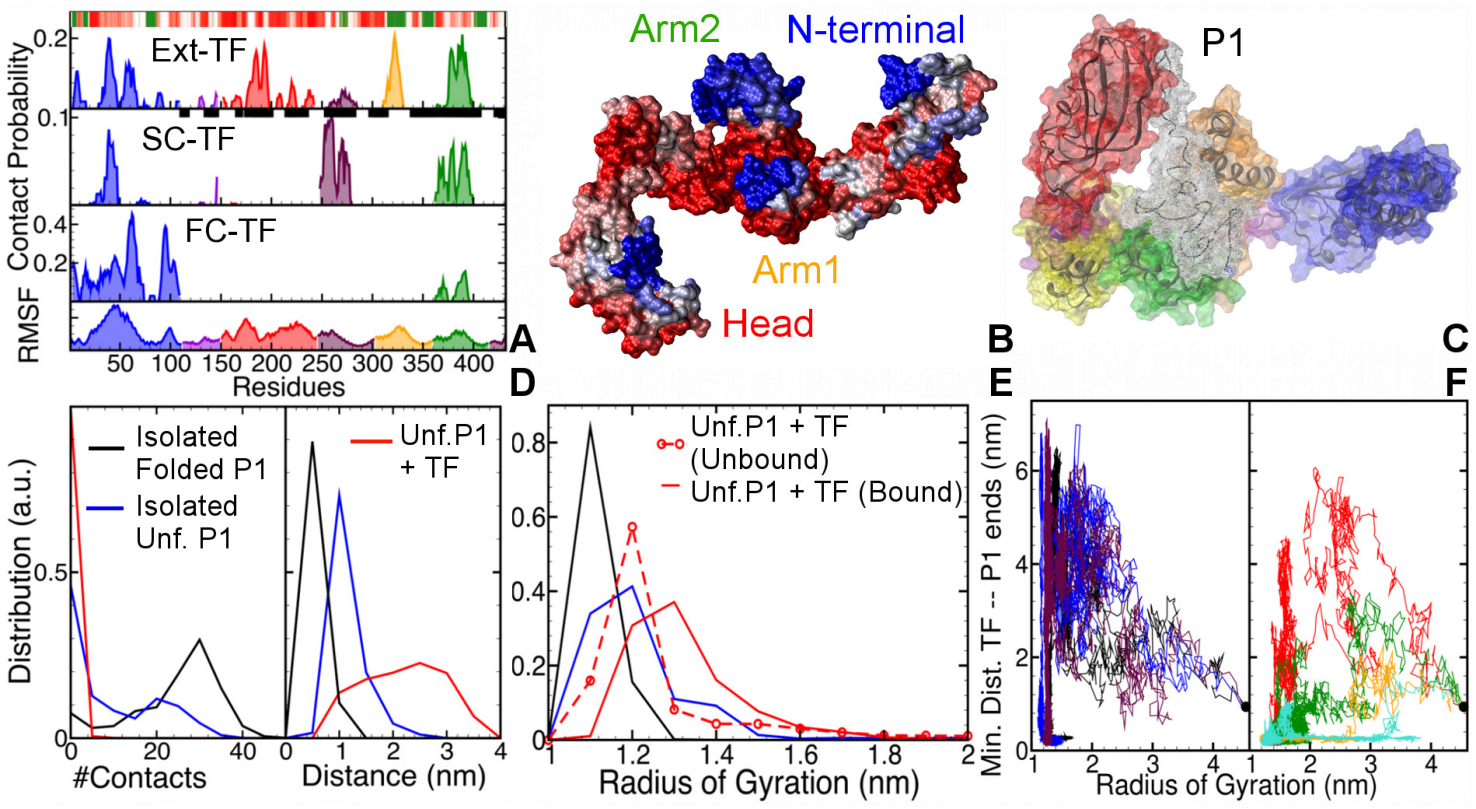
**A.** Contact probabilities of the residues of the extended (Ext), semi-collapsed (SC) and fully collapsed (FC) conformations TF with unfolded P1. The bottom-most panel plots the root mean squared fluctuations of TF’s residues, with peaks indicative of flexible regions. Colors refer to the different TF domains: N-terminal in blue, Linker in violet, PPIase domain in red, HA1-linker in maroon, Arm1 in orange, and Arm2 in green. The black squares on the residue (x-) axis of the extended TF plot shows the interaction sites identified by Saio, *et al*. [14]. **B.** Visualization of the most likely interaction sites (blue) on TF’s surface. **C.** Final TF-P1 complex obtained from one of the simulations, with shown in transparent white colored wire-mesh. **D.** Distribution of end-to-end contacts (left panel), and COM distance between the first and last 5 residues (right panel) in P1. **E.** Distributions of the radius of gyration of P1. **F.** 2-D plots of minimum distance between TF and P1’s ends against radius of gyration of unfolded P1, when: P1 collapses before the contact formation (left panel) and after contact formation (right panel) with the P1’s ends.

Fig 2A plots the contact probability for TF’s residues with unfolded P1 (In this work we represent the tendency of interaction by contact probability rather than the interaction free energy, because of the non-equilibrium nature of the simulations). The bottom-most panel shows the root-mean square fluctuations of the C-*α* atoms of TF residues. The extended TF’s interactions with unfolded P1 are non-specific and distributed over the entire surface of TF, with strong interaction sites at the N-terminal (residues 38–42), Head (residues 184–186 and 192–194), Arm1 (residues 320–325) and Arm2 (residues 378–390). The strongest contacts are made at hydrophobic residues, viz., Phenylalanine, Isoleucine and Tyrosine. The flexible tip of Arm1, which also contains a strongly hydrophobic patch, hosts the strongest interaction site. Because the PPIase domain is bound to the Arm1 in the semi-collapsed and fully-collapsed conformations of TF, both these domains are unavailable for substrate binding. Consequently, there are no interaction sites on PPIase domain and Arm1 in the SC and FC conformations. They are replaced by new sites on Arm2 (in particular, *Lys*368) and HA1-Linker (residues 254–275) in P1’s association with semi-collapsed TF. In case of the fully-collapsed TF, the mostly hydrophilic external surface of N-terminal dominates the interaction sites with the substrate. Fig 2B maps the contact probability on an extended TF structure. Most of the interaction sites (blue) lie at the tips of various domains, except for those on the Head, which covers the attached chain with its inner surface, like a lid.

Our results corroborate the findings of Lakshmipathy *et al*. [36, 37] and Saio *et al*. [14], who showed that TF employs both arms—especially the flexible loops at the tips of the arms—and N-terminal to bind nascent polypeptide chains. Fig 2A compares the interaction sites estimated in this work with those reported by Saio, *et al*. [14]. The interacting residues identified by Saio *et al*. are represented by black squares on the x-axis of top panel (Please note that Saio *et al*. did not provide information on the relative strengths of these interaction sites). Almost all of the interaction sites identified in their work coincide with the peaks of the contact probability map found in our simulations, especially at the tips of the arms and inner surface of Head. However, our simulations did not capture the relatively long process of TF fully encapsulating the unfolded substrates, and hence, the interaction sites in the cleft of the cradle—between residues 350 and 370. Instead, our simulations highlight the events in the early interaction dynamics, where TF binds unfolded substrates with the tips of its appendages (including N-terminal), and subsequently collapses around them to strengthen the interactions.

To investigate how the early interactions with TF affect the unfolded P1, we compared its dynamics in complex with extended TF with that of unfolded P1 in isolation. We focused on the effect of TF on the ends of P1, which are in a strong direct contact with each other in the folded state. We observed that in the presence as well as absence of TF, the unfolded P1 collapses into a molten globule with a small radius of gyration, comparable to that of folded P1. This spontaneous collapse brings P1’s ends together, leading to the formation of strong transient non-native contacts, which can result in misfolded states. TF slows down this spontaneous collapse and largely prevents the formation of non-native end-to-end contacts in P1. This is evident in the distribution of the number of P1’s end-to-end contacts, plotted in the left panel of Fig 2D. The presence of TF shifts this distribution to lower values w.r.t. the unfolded P1 in isolation, resulting in almost zero end-to-end contacts in P1. In contrast, the distribution for folded P1 is narrower than unfolded P1 and peaks at a higher number of contacts. The right panel of Fig 2D shows that the distribution of the end-to-end distance in P1 in complex with extended TF is wider and shifted to larger distance w.r.t. P1 in isolation. The distribution for folded P1 in isolation has a sharp peak at an even lower value of 0.5 nm. An increase in the end-to-end distance in presence of TF translates into a higher radius of gyration of P1 (see Fig 2E). The unbound P1 in isolation and in the system with TF have comparable compactness. Upon association, the distribution shifts to higher radius, implying that TF not only hinders collapse, but also stretches collapsed P1, likely avoiding bad intramolecular contacts and facilitating sampling of larger conformational space (see S2 Fig). Fig 2F plots the minimum distance TF and P1’s ends versus the radius of gyration of P1 for all seven trajectories that demonstrate binding. Unfolded P1 can either first collapse and then bind TF (left panel), or vice-versa (right panel). Strikingly, in both cases P1 opens up after associating with TF, and samples higher values of radius of gyration. The highest values are sampled in the trajectories where binding occurs before the collapse. One of the final TF-P1 complexes, shown in Fig 2C, demonstrates a relatively open conformation of P1, held by the Head and the tips of the arms. The deformed conformation of TF suggests that extended TF adapts to the substrate size and structure (see S2 Fig).

These results point to a mechanism for the chaperone action of TF, wherein TF: 1) binds to an unfolded chain; 2) prevents the formation of spontaneous transient, non-native end-to-end contacts; 3) slows down the chains collapse; and, 4) facilitates the chain to explore more conformations than the unfolded chain can in isolation.

### TF and Folded P1

Mashaghi *et al*. found recently that TF binds partially folded structures formed in MBP chains [18]. In order to elucidate the structural basis for this binding, we performed MD simulations of folded P1 in the presence and absence of TF (starting in the extended conformation). We found that folded P1 indeed forms a complex with TF, though this requires more time than for unfolded P1, and involves more frequent detachments (Table 2). Semi-collapsed and fully-collapsed conformations of TF display shorter mean first binding times and fewer detachment events, even slightly smaller compared to unfolded P1. This may be because these TF conformations preferably bind to the near-folded substrates or folding intermediates in cytosol to isolate them.

**Fig 3:**
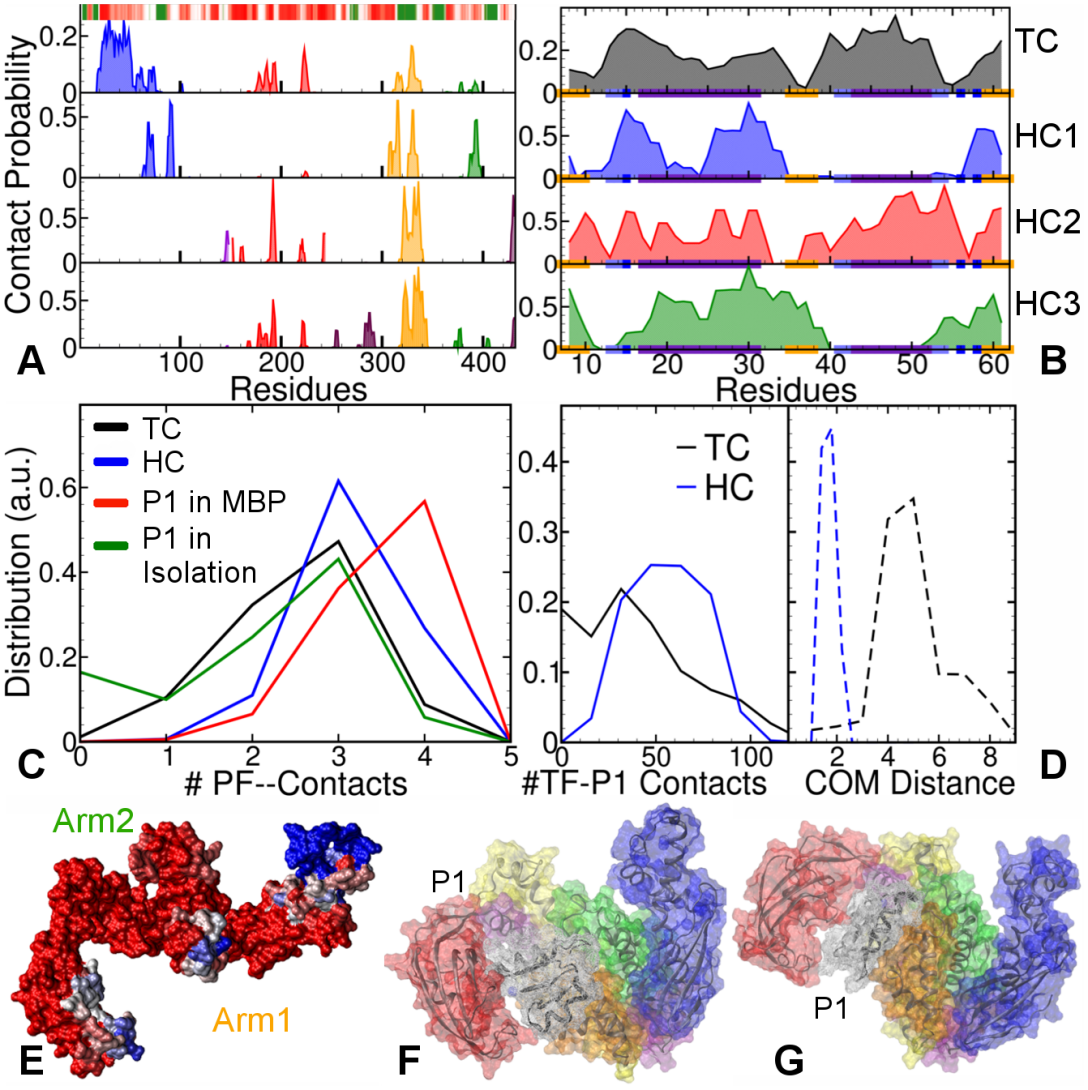
Comparisons between MD simulations of folded P1 with TF in Touching Complex (TC), and Hugging Complexes (HCs). **A.** Contact probabilities of TF residues with P1 in four TF-P1 Touching and Hugging Complexes. The barcode plots the hydrophobicity map of TF: color-scaled from red (hydrophilic) to green (hydrophobic). **B.** Corresponding contact probabilities of P1 residues with TF plotted in the same order of panels. The secondary structure of P1 is represented on the x-axis: orange regions for *β*–strands, indigo for *α*–helices, iceblue for turns, dark blue for bends, and the rest represents coils. **C.** Distributions of the number of intact PF-contacts in folded P1. **D.** Distributions of the number of TF-P1 contacts (left panel) and TF-P1 COM distance (right panel), in Touching Complexes(black lines) and Hugging Complexes (blue lines), respectively. **E.** Visualization of the interaction sites on TF’s surface the P1 in the TC, colored from red (least likely) to blue (most likely). **F.** Representative final TC conformation, with P1 shown in transparent white colored wire-mesh. **G.** Representative final HC conformation.

The top panel (TC) of Fig 3A shows contact probabilities of TF residues with folded P1 in the extended TF simulations. Most of the interaction sites lie on the largely hydrophilic N-terminal (residues 24–32 and 45–54), and only a few on Head and hydrophobic Arm1, indicating that folded P1 explores fewer sites than the unfolded P1 (Fig 2A). The corresponding contact probabilities of the P1 residues are plotted in the top panel of Fig 3B. The interaction sites are uniformly distributed over the entire surface of P1, including the ends. For extended TF, most of the interaction sites (blue) are located on the flexible loops at the tips of N-terminal (residues 30–50) and Arm1 (Fig 3E). Similar analysis (see S3 Fig) for the semi-collapsed and fully-collapsed conformations of TF reveals that folded P1 attaches primarily to the N-terminal, and that the other domains rarely interact with folded P1. Thus, TF initially interacts with folded P1 via the tips of one or two of its appendages, dominated by N-terminal. We therefore refer to these complexes as “Touching Complexes” (TC).

With the aim to probe the post-folding dynamics of P1’s interactions with TF, we initiated MD simulations using the lowest energy configurations for rigid TF and P1 obtained from docking calculations (see Methods). We found that in these simulations, P1 moved closer to the cradle center, and was encapsulated by the flexible TF appendages, led by Arm1. Therefore, we referred to these structures as “Hugging Complexes” (HC) (see S4 Fig). The contact probabilities for the P1 residues in these simulations (plotted in the lower three panels in Fig 3B) are less uniform than those in the TC. The corresponding three panels for TF in Fig 3A show strong interaction sites with Arm1 (yellow) for all HCs. These interaction sites also occur in TC, indicating the central role of Arm1 in substrate binding. P1 typically interacts with two TF elements: either with Arm1 and PPIase, or with Arm1 and the N-terminal. Fig 3G shows a representative final HC conformation (HC2), where P1 binds at the Arm1 and PPIase domain. This complex is similar to the final TC conformation shown in Fig 3F, suggesting an (partial) overlap between the conformational space of the TC and HC simulations.

To investigate whether TF-binding stabilizes P1, we monitored the number of intact (out of four) *β*-sheet hydrogen bonds between the strands formed by residues 7–11 and 58–62, referred to as “partial fold” (or PF) contacts. Probability distributions for these contacts are plotted in Fig 3C. In the full MBP, these hydrogen bond contacts are almost all intact, stabilized by the surrounding structure of MBP. In contrast, the truncated P1 in isolation is less stable and shows loss of PF-contacts, with even a (small) peak at zero contacts. The binding of P1 with TF in Touching Complexes stabilizes it w.r.t. P1 in isolation, as illustrated by the loss of the peak at zero contacts. Hugging Complexes show more intact contacts than the TC. This is consistent with the increased proximity of P1 to the TF’s cradle in the Hugging Complex, as indicated by the corresponding probability distributions of the center of mass (COM) distance between two molecules in Fig 3D (dashed lines). The probability distribution for the number of TF-P1 contacts shown in the same figure (solid lines), indicates that the increased proximity results in a higher number of TF-P1 contacts. Thus, after or during folding, P1 can move towards TF’s interior, which increases the stabilization of its PF-contacts. This mechanism is reminiscent of the “cradle model” for TF’s function, which speculates that the protein folding and stabilization happen in the cradle-like interior of TF [10].

**Fig 4:**
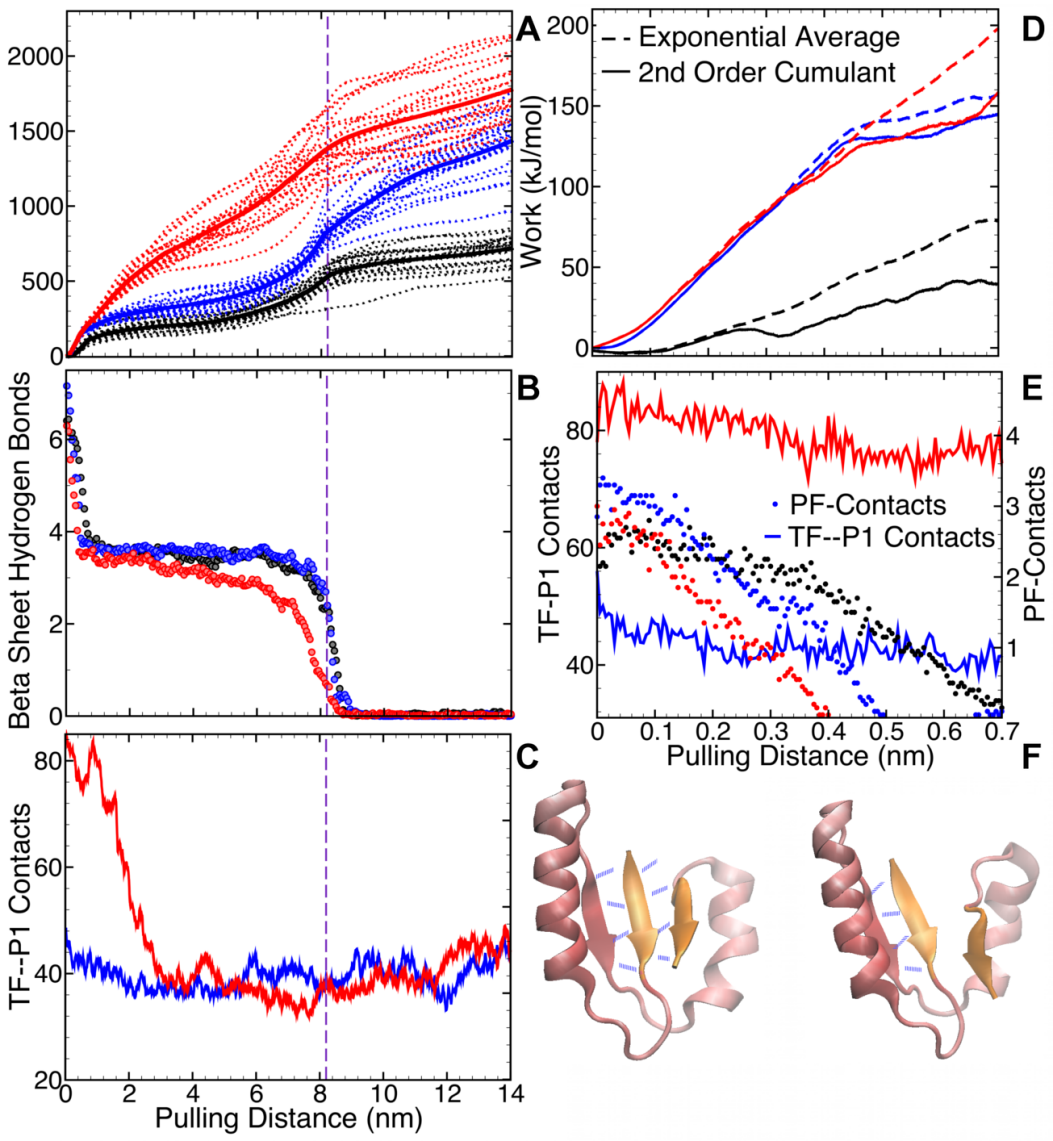
Steered MD simulations of pulling the folded P1 apart to unfold it in isolation (black lines), and in complexes with TF: HC1 (blue lines) and HC2 (red lines). **A.** Work-extension graphs of each trajectory (dashed lines) and average work (bold solid lines); **B.** Average number of P1 *β*–sheet hydrogen bonds in the three systems; **C.** Average number of TF-P1 contacts. The vertical dashed lines at pulling distance of 8.2 nm marks the end of the energy barrier. **D.** Plots of second order cumulant expansion (solid lines) and Boltzmann-weighted average work plot (dashed lines) for each system in the first 1.4 ns or over 0.7 nm of pulling. **E.** The PF-contacts (circles) are broken while TF-P1 contacts (solid lines) remain almost unchanged in the first 1.4 ns (or 0.7 nm) of pulling. **F.** Cartoon representation of the loss of 4 hydrogen bonds between the first and last *β*–strands (colored orange) of P1 upon pulling.

A comparison between the unfolding potential of mean force (PMF) of folded P1 in isolation using steered MD (SMD), and that of P1 in Hugging Complexes (S5 Fig) provides more insight into the nature of TF’s stabilization of P1. As the interactions maps in the HCs 2 and 3 are comparable, we selected the final configuration of the MD simulation of HCs 1 and 2 as input for the SMD. Pulling at the ends (first and last residues) of folded P1, we measured the cumulative work required to unfold P1 (see Fig 4A). We found that, compared with P1 in isolation, a higher amount of work is needed to unfold P1 in complex with TF. Fig 4B indicates that the loss of P1’s *β*–sheet network is the primary contributing factor in the free energy barriers in the pulling process. The *β*–sheet network is formed by two sets of backbone hydrogen bonds: those between residues 7–11 and 58–62, and those between residues 7–11 and 35–39. There are, on average, four hydrogen bonds between each pair of *β*–strands, and the sudden loss of each set coincides with a jump in work. The changes in TF-P1 contacts (Fig 4C) do not seem to contribute to the free energy barriers.

We used Jarzynski’s inequality [30], and the second cumulant approximation [31, 38] to turn the non-equilibrium work-extension curves into equilibrium free energy. In the early unfolding regime—extension of 0.7 nm over 1.4 ns of pulling, the exponential average of work and the second order cumulant expansion coincide (Fig 4D), which confirms the reliability of PMF in this regime [31, 38]. For the complete unfolding process, however, the variance in the work-extension graphs was too large, making the resulting PMF unreliable. For this reason, we focused only on the early unfolding regime, which contains the first major free energy barrier in all three systems, caused by the loss of PF-contacts (shown by circles in Fig 4E). The energy barriers in both the TF-P1 systems are significantly bigger than that for P1 in isolation. Breaking these PF-contacts, thus, requires more energy when P1 is in complex with TF than in isolation. The number of TF-P1 contacts (solid lines) decrease by ∼ 10 in both HC1 and HC2. This loss may also contribute to the amplification of the free energy barrier. Fig 4F visualizes the loss of PF-contacts through breaking of hydrogen bonds between the first and last *β*–strands (colored orange) upon pulling.

To summarize, TF stabilizes protein’s partial folds by encapsulating them in its interior (and adapting to their structure), and strengthening their PF-contacts.

### P2 and Full MBP

**Fig 5:**
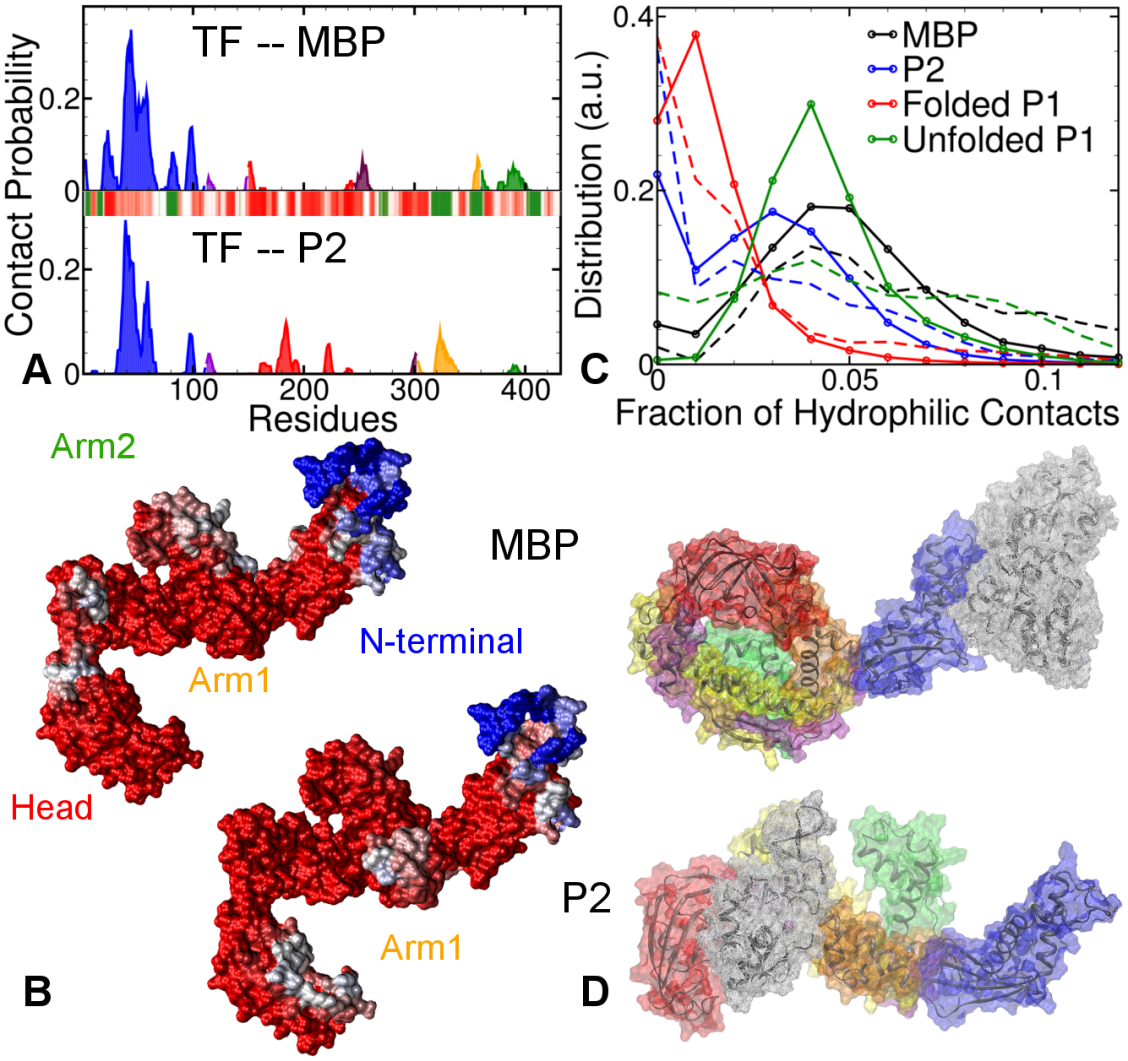
Four 200 ns long AA-MD simulations of extended TF each with full MBP and P2. **A.** Contact probabilities of TF residues with MBP (top panel) and P2 (bottom panel). The barcode plots the standard hydrophobicity map of TF: color-scaled from red (hydrophilic) to green (hydrophobic). **B.** Distribution of the fraction of hydrophilic contacts in TF-substrate binding for different substrates. The dashed lines plot the distribution in the first 10 ns, while solid lines with circles plot the distribution over the whole trajectory. **C.** Visualization of the most important interaction sites (blue) on TF for MBP and P2. **D.** Representative final conformations of TF-MBP and TF-P2 complexes. The substrates are shown in transparent white colored wire-mesh.

Once the smallest partial-fold (P1) is completely folded, TF further interacts with the larger partial folds (e.g., P2), eventually interacting with full MBP. We simulated these interaction through six MD runs of TF with a larger partial fold P2, and four runs with full native MBP molecule (see S6 and S7 Figs). The contact probability plots in Fig 5A (top panel for MBP, bottom panel for P2) show that for both systems, the tip of N-terminal domain (residues 38–55) is responsible for the majority of the interactions, while other domains play only minor roles. P2 also binds to the tips of Arm1 (residues 320–325). The TF structures in Fig 5B visualize the location of these interaction sites with MBP and P2. Most of them lie at the tips of TF’s appendages, while the PPIase domain again appears to cover P2, like a lid, with its inner surface. The hydrophobicity maps show that the dominant interaction sites for both substrates lie in hydrophilic regions. Fig 5D visualizes the TF-substrate binding with two representations of TF-MBP and TF-P2 complexes.

Both substrates demonstrated association and dissociation events with TF in all simulations. The mean first binding time as well as the number of detachment events for P2’s binding with TF were comparable with those of P1’s binding with extended TF (Table 2). Native MBP, on the other hand, detaches more often over a longer duration before it forms an apparently metastable complex with TF. The stability of TF-substrate complexes increases for substrates with lower degree of structure, i.e., native MBP interacts weakly with TF relative to the partial folds, which in turn form complexes less readily than the unfolded substrates. These findings match the observations of Mashaghi *et al*. [18], who showed that native MBP does not bind to TF as strongly as its partial folds do. This suggests that as the MBP folds, its binding with TF progressively weakens, which can help in the post-folding release of the protein from TF.

To investigate the variation in the nature of TF’s interactions with different partial folds, we compared the ratio of hydrophilic contacts to total contacts, between TF and different substrates (Please note that this estimate has a bias towards a large number of mixed contacts, i.e., Oxygen and Nitrogen atoms with Carbon atoms, and is therefore conservative). Fig 5C plots the distribution of these ratio in the first 10 ns of contact (dashed lines) and during the entire duration of contact (solid lines with circles). Overall, the TF-MBP interactions are most hydrophilic, followed by extended TF-unfolded P1 and TF-P2 interactions, with extended TF-folded P1 interactions being the most hydrophobic. The small positive shifts in this ratio for folded P1 and P2 after the first 10 ns indicates that for partial folds, hydrophobic contacts initiate the binding, which becomes gradually more hydrophilic. In case of unfolded P1, the distribution in the first 10 ns is wider and lower, while the overall distribution has a distinct sharp peak comparable to full MBP. This suggests that the large available surface area in unfolded P1 enables both hydrophobic and hydrophilic contacts to initiate the binding with TF.

### Summary

In a recent study, the chaperone Trigger Factor was shown to direct Maltose Binding Protein (MBP) chains to their native state by transiently stabilizing specific partially folded intermediate states [18]. These findings allowed us to undertake an all-atom molecular dynamics simulation study of the interaction between Trigger Factor and the MBP protein chain in its different stages of folding.

We found that TF interacts with folded structures with the ends of its flexible appendages, especially N-terminal, thus forming a “Touching Complex”. The residues 45–54 (tip of N-terminal) and 24–32 (also N-terminal) interact most frequently with the substrates. During the interaction with the substrates, TF’s secondary structure remains intact, while its tertiary structure can adapt to the substrates, as indicated by structural deviations in the flexible linker regions (see Supporting Information). TF can leverage this flexibility to wrap its appendages around the folded structures (P1), forming the more stable “Hugging Complexes,” while other MBP segments remain unfolded.

Our steered MD simulations showed that breaking the end-to-end *β*–sheet hydrogen bonds of P1 in these complexes requires more work than when P1 is in isolation. This suggests that by wrapping around partially folded structures and establishing multiple favorable contacts, TF stabilizes these partial folds. The flexible appendages and the heterogeneous nature of its surface allow TF to bind to different intermediate folds of the same protein, and, by induction, indeed to proteins of different sizes. This binding is the strongest with unfolded protein chains, and weakens gradually with the progressive folding of the polypeptide. It weakens significantly upon the formation of fully folded MBP, which likely plays a role in the post-folding release of the protein.

The unfolded chain binds simultaneously with two to three sites on TF, in particular with the flexible loops at the tips of N-terminal (residues 38–40), Arm1 (residues 320–325) and Arm2 (residues 378–380 and 387–390). In TF’s interactions with nascent polypeptide chains, the tips of the arms were found to be important binding sites in the NMR experiments by Saio, *et al*. [14], as well as the fluorescence spectroscopy measurements by Lakshmipathy *et al*. [36, 37]. This consistency validates the relevance of interactions predicted by our extensive molecular simulations.

It has been observed that the Arm1 plays an important role in TF’s collapse by strongly interacting with the PPIase domain [12, 13]. Consequently, Thomas, *et al*. speculated that the TF’s collapse may adversely affect its interactions with unfolded chains. Our findings confirm this suggestion—the binding of unfolded polypeptides is indeed much weaker with the collapsed conformations of TF than with the extended conformation, presumably due to the lack of Arm1’s availability in the former.

We found that unbound to TF, unfolded P1 collapses spontaneously into a molten globule with the formation of strong non-native contacts, which can lead to misfolding. In contrast, TF slows down, and even reverses, this collapse, thus allowing the unfolded chain to explore a larger conformational space than in isolation. We can, thus, attribute TF’s role in protein folding to creating kinetic traps in the formation and, subsequently, breaking of the intramolecular contacts of the protein’s folding intermediates in order to prevent misfolding and aggregation. Employing coarse-grained MD on TF-ribosome complex, O’Brien *et al*. have suggested a similar chaperone action for TF at the ribosome [19].

**Fig 6:**
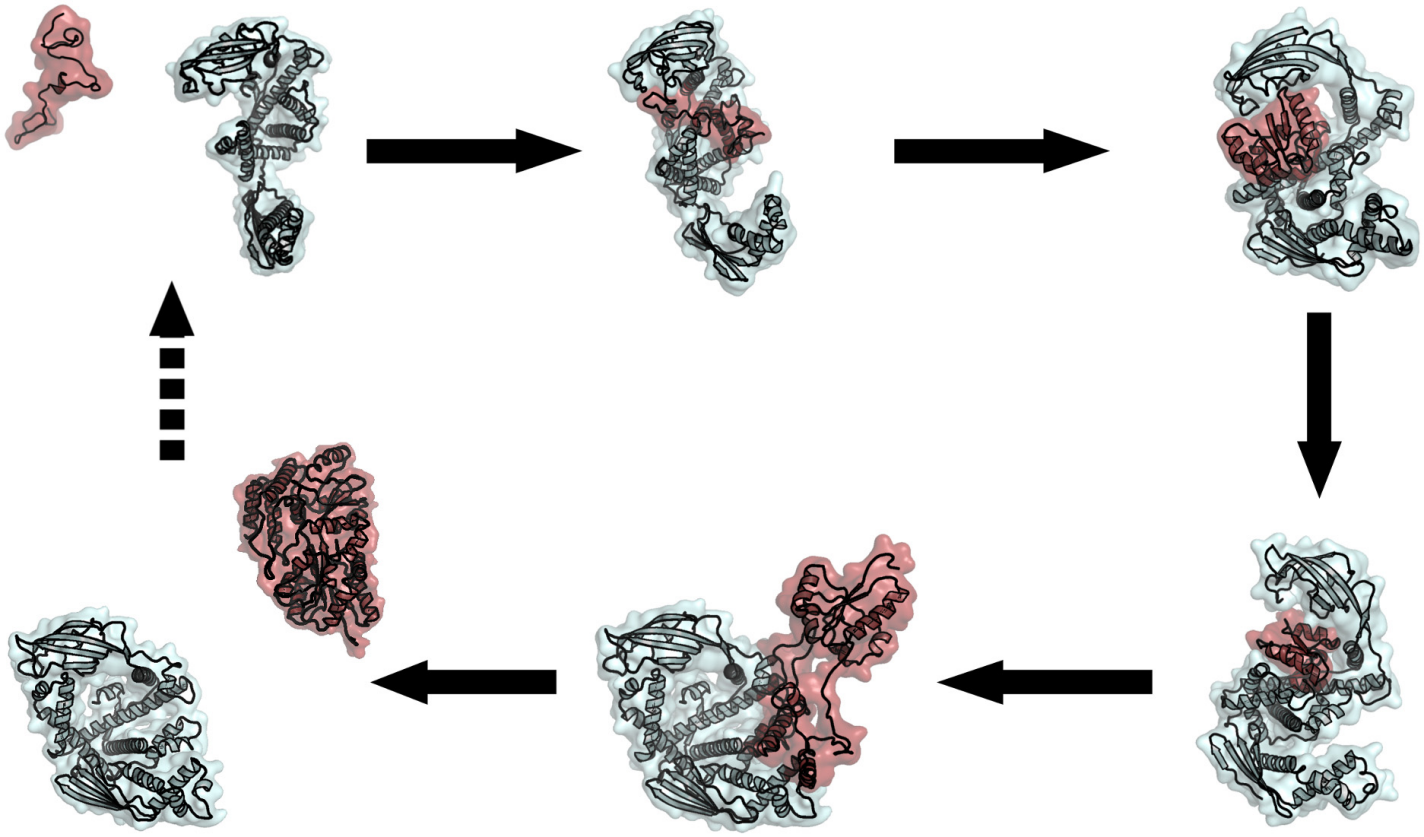
Cartoon representation of the folding of MBP in complex with TF. For each folding stage, a representative snapshot is shown, with MBP (and its partial folds) in pale red and TF in cyan. The schematic shows a series of events in the cycle, which starts with the interactions between folding intermediates of substrate proteins (MBP in this case) with TF and results in the fully folded substrate protein released from the TF at the end. This may involve more than one TF molecules.

Together with the previous findings, our results suggest the following scenario, which is summarized in Fig 6. TF employs the flexible loop at its domain tips (mostly its arms) to bind a small part of the MBP chain. As the TF transfers this part of the chain into its cradle, the chain forms (either simultaneously or subsequently) a partial fold. TF then shields and isolates this partial fold from the rest of the protein, and continues to fold and stabilize intermediates of increasing size until the native state of MBP is reached. The weak binding of native MBP with TF facilitates its subsequent release into the solution. In light of the recent work by Saio, *el al*. [14], it is likely that more than one TF monomer may be involved in subsequent folding of MBP. This model provides a structural explanation for the ability of highly adaptable TF chaperone to interact with and stabilize a wide spectrum of substrates, ranging from unfolded chains to the diverse intermediate folded structures *en route* to the native state [18]. By providing a flexible encapsulating cradle, TF protects unfolded and partially folded chains against misfolding interactions with other domains of MBP or other proteins.

We realize that the use of truncates and short trajectories cannot represent the full folding, binding and chaperone action, and that atomistic force fields are not perfect. Nevertheless they can give additional atomistic insight. Among the different atomistic force fields Amber03 and Amber99SB-ILDN, especially the latter, are perceived to be reasonably good for protein folding and unfolding transitions [24]. However, a full comparison between different force fields is beyond the scope of this study.

Regardless, this work provides the first atomistic view on a chaperone-client complex in different stages of folding, offers an (atomistic) explanation for the ability of TF to guide chains to their native state by stabilizing partial folds and protecting them against misfolding and aggregation interactions. It builds on the previous work by Saio, *el al*. [14] and O’Brien *et al*. [19], and confirms that TF’s flexibility is central to its ability to interact with a wide range of client states. While it lends a new hypothesis about the Trigger Factor MBP system in particular, it also yields generic insights into how an ATP-independent chaperone can assist folding and prevent misfolding.

There are many questions that still need to be explored. For example, how does TF distinguish partially folded structures from fully folded structures? It is not clear how precisely the substrate transitions from one state to the next: does TF completely detach or remain attached as the substrate progressively folds? Do other chaperones exploit similar mechanisms? We expect advanced molecular simulation methods, along with increasing computer power, to complement improving experimental techniques in answering these questions in a very near future.

## Supporting Information

S1 Text Metastability of complex formation. We performed multiple MD simulations of several TF-substrate systems. In most TF-substrate simulations, we observed diffusive behavior, with many spontaneous attachment events between TF and the substrate via both hydrophilic and hydrophobic interactions, as well as detachment events. This text analyzes them in greater detail.
(PDF)

S2 Text Additional structural analysis. In addition to the analysis presented in the main text, this section provides further information on the structural dynamics of chaperone-protein complexes. It also shows the final conformations of the different extended TF-substrate complexes acquired from the MD simulations and Rosetta docking calculations.
(PDF)

S1 Fig Minimum distance between the heavy atoms of TF and: **A.** MBP (top panel) and P2 (bottom panel); **B.** extended, semi-collapsed and fully-collapsed conformations of TF with Unfolded P1; **C.** extended, semi-collapsed and fully-collapsed conformations of TF with Folded P1; **D.** Distributions of final radii of gyration of TF in complex with different substrates. Black graph plots the same for TF in isolation.
(TIF)

S2 Fig **A.** Starting and final (7) configurations of extended TF–Unfolded P1 simulations. P1 is shown in transparent white colored wire-mesh. **B.** 2-D projection along first two eigenvectors from dPCA of the combined dynamics of unfolded P1 in presence (blue) and absence (black) of TF. Unfolded P1 explores a larger conformational space in complex with extended TF than in isolation. **C.** Changes in the distribution of solvent accessible surface (SAS) area of unfolded P1 from the first 10 ns to the last 10 ns in presence of extended TF. Green graph plots the SAS area of folded P1 in absence of TF. **D.** Contact probabilities of the Unfolded P1 residues with extended, semi-collapsed and fully-collapsed conformations of TF. Barcode on the x-axis shows the supposed secondary structure of folded P1: orange regions for *β*–strands, indigo for *α*–helices, iceblue for turns, dark blue for bends, and the rest represents coil.
(TIF)

S3 Fig **A.** Starting and final (4) configurations of extended TF–Folded P1 simulations, forming “Touching Complexes.” P1 is shown in transparent white colored wire-mesh. **B.** Contact probabilities of the TF residues with Folded P1 in extended, semi-collapsed and fully-collapsed conformations of TF.
(TIF)

S4 Fig **A.** Starting configurations of “Hugging Complexes” (folded P1 docked on extended TF) as obtained from Rosetta docking calculations. **B.** Final configurations of the complexes after 100 ns of MD simulations. P1 is shown in transparent white colored wire-mesh. Time evolution of the **C.** centre of mass distance and **D.** number of contacts between TF and folded P1 over 100 ns.
(TIF)

S5 Fig Representative structure of: **A.** System1 and **B.** System2, before and after pulling. Purple beads show the points at which the opposing pulling forces are applied. Change in the fraction of native C-*α* contacts of P1 over **C.** 15 nm of pulling that leads to unfolding. **D.** 1st 0.7 nm of pulling that leads of breaking of PF-contacts. Also plotted on the same time scale are the average work-extension graphs. Black graphs represent P1 in isolation, blue graphs show P1 in System 1, and red graphs show P1 in System 2.
(TIF)

S6 Fig **A.** Starting and final (6) configurations of extended TF–P2 simulations. P2 is shown in transparent white colored wire-mesh. **B.** Contact probability of the P2 residues with extended conformation of TF. Barcode on the x-axis shows the supposed secondary structure of P2: orange regions for *β*–strands, indigo for *α*–helices, and the rest represents coil, bends and turns.
(TIF)

S7 Fig **A.** Starting and final (4) configurations of extended TF–MBP simulations. MBP is shown in transparent white colored wire-mesh. **B.** Contact probability of the MBP residues with extended conformation of TF. Barcode on the x-axis shows the supposed secondary structure of MBP: orange regions for *β*–strands, indigo for *α*–helices, and the rest represents coil, bends and turns.
(TIF)
