# Supplementary material for: The Trigger Factor Chaperone Encapsulates and Stabilizes Partial Folds of Substrate Proteins
Source: PLoS Comput Biol. 2015 Oct 29;11(10):e1004444. doi: 10.1371/journal.pcbi.1004444 (PMC4626277; doi:10.1371/journal.pcbi.1004444)
Supplement: S1 Text — We performed multiple MD simulations of several TF-substrate systems. In most TF-substrate simulations, we observed diffusive behavior, with many spontaneous attachment events between TF and the substrate via both hydrophilic and hydrophobic interactions, as well as detachment events. This text analyzes them in greater detail. (PDF) [file pcbi.1004444.s001.pdf]

S1 Text: Metastability of complex formation

We performed multiple MD simulations of several TF-substrate systems (See Methods). In most TF-substrate simulations we observed diffusive behavior, with many spontaneous attachment events between TF and the substrate via both hydrophilic and hydrophobic interactions, as well as detachment events. (Note that we do not use the words *binding* and *unbinding*, as these are reserved for the full equilibrium process of complex formation, whereas here we only observe the initial stages of binding). Table 2 (in the main text) gives the frequency of these events and the dissociation constant as estimated from the ratio of the detached to the attached time. The dissociation constant is indicative of the metastability of the complex, whereas the frequency is determined by the height of the attachment and detachment free energy barriers. The dissociation constant naturally is a function of volume (or substrate concentration). For completeness, we also included the attachment and detachment rates per ns. While the error-bars on these numbers are necessarily large, we can still draw some qualitative conclusions.

For the extended TF the frequency of detachment events decreases significantly from native MBP to P2 and folded and unfolded P1. The dissociation constant also shows roughly the same trend. The dissociation rate of MBP is higher than that of P2 and P1, meaning that full MBP has weaker interactions with TF compared to P2 and P1. The association rate of MBP is also higher than that of P2 and P1, probably due to the size of the full protein and the larger number of possible interaction sites in full MBP. However, for folded P1 with extended TF we observe a similar (or even a bit higher) attachment rate as for P2, suggesting that size is not the only factor determining TF-substrate complex formation. The folded P1 appears to have more difficulty forming metastable complexes with SC- and FC-TF conformations than with extended TF (with significant lower attachment/detachment frequency and association rates). The barriers involved in the attachment/detachment process are higher, since the collapsed TF structures have less interaction sites available.

The number of detachment events is very low for the system with unfolded P1 and the extended structure of TF, but increases for unfolded P1 with SC- and FC-TF conformations. The dissociation and the association rates show same trend. Interestingly, for SC- and FC-TF conformations the association and dissociation rates with the unfolded P1 are higher than for the folded P1. This means that the involved barriers are actually lower for the unfolded P1. Indeed, this is what one would expect from a chaperone as it suggests a selection mechanism of TF in which unfolded chains are more easily captured

than folded ones.

Clearly, the stability of extended TF-substrate complexes increases for substrates with lower degree of structure, i.e., native MBP interacts weakly with TF relative to the partial folds, which in turn form metastable complexes less easily than the unfolded substrates. This is in agreement with the observations of Mashaghi *et al.* [**?**], who showed that native MBP does not bind to TF as strongly as its partial folds do. This suggests that as the MBP folds, its binding with TF gradually weakens, which likely helps in the post-folding release of the protein from TF.
