# Supplementary material for: The Trigger Factor Chaperone Encapsulates and Stabilizes Partial Folds of Substrate Proteins
Source: PLoS Comput Biol. 2015 Oct 29;11(10):e1004444. doi: 10.1371/journal.pcbi.1004444 (PMC4626277; doi:10.1371/journal.pcbi.1004444)
Supplement: S2 Text — In addition to the analysis presented in the main text, this section provides further information on the structural dynamics of chaperone-protein complexes. It also shows the final conformations of the different extended TF-substrate complexes acquired from the MD simulations and Rosetta docking calculations. (PDF) [file pcbi.1004444.s002.pdf]

S2 Text: Additional Structural Analysis

In addition to the analysis presented in the main text, this section provides further information on the structural dynamics of chaperone-protein complexes. It also shows the final conformations of the different extended TF-substrate complexes acquired from the MD simulations and Rosetta docking calculations.

In nearly all MD simulations, TF interacts with the different substrates – MBP, P2, unfolded P1 and folded P1. The proteins are said to be in contact when the minimum distance between the heavy atoms of the respective TF conformation and the substrate decreases to less that 0.4 nm. Figure S1 shows that the respective minimum distances between the proteins indeed fall below 0.4 nm in almost all cases. Owing to the small size of the P1, it is able to diffuse around the box without finding a stable binding site on TF more easily than larger substrates. Figures S1B and C plot the minimum distances between the three conformations of TF – extended (Ext-TF), semi-collapsed (SC-TF) and fully-collapsed (FC-TF) – and unfolded P1 and folded P1 respectively. For each conformation of P1, there is one trajectory that does not show stable binding with TF. This is most likely a statement on the large amount of simulations performed with P1 than P1's inability to bind with TF.

The adaptability and flexibility of TF is a strong theme in this work, and in literature. We address this flexibility here in terms of the change in TF's compactness (starting from its extended conformation) while in complex with substrates of different sizes. Figure S1D plots the distribution of the radii of gyration of TF when: in isolation (black graph), in complex with MBP, in complex with P2, in complex with folded P1 (touching complexes), in complex with folded P1 docked on it (hugging complexes), and in complex with unfolded P1. The dashed magenta line indicates the starting radius of gyration of TF. In hugging complex and in complex with unfolded P1, TF was more compact than even when in isolation. The distributions are wider and shifted to higher values for TF in complex with MBP and P2, which are bigger than both unfolded and folded P1. This indicates that TF is highly flexible and can adapt to the size and shape of the substrate.

Figure S2A shows the starting and the seven final conformations of TF in complex with unfolded P1. In all final complex conformations, unfolded P1 collapsed into a compact globular conformation, while TF collapsed around P1 and adapted to P1's structure. We suggested in the paper that P1 explores a larger conformational space in the presence of TF than it does in isolation. To support that suggestion, figure S2B plots the conformational dynamics of unfolded P1 in isolation (black graph) and in complex

with extended TF (blue graph). The conformational landscape is defined by the two largest eigenvectors obtained from the principal component analysis of the combined structural dynamics of P1 in isolation and in complex with TF (see Methods). It is evident that in this conformational space, the slowing down of the dynamics by TF helps unfolded P1 sample a larger area.

In complex with TF, P1's solvent accessible surface (SAS) area decreases, led by the drop in hydrophobic surface area. This phenomenon is shown in figure S2C, which plots the distributions of the total, hydrophobic, and hydrophilic SAS areas of P1 in complex with TF. The solid lines represent the distributions in the first 10 ns, while the dashed lines shows the distributions in the last 10 ns of simulations. Green graph shows the distribution of the total SAS area of folded P1 in isolation, which is comparable to that of unfolded P1 in the first 10 ns of the simulations. In the main text, we presented the contact probability maps of the interaction of TF's residues with unfolded P1, starting with different conformations of TF. It may be worthwhile to look for specific patterns in P1's interaction with different conformations of TF. Figure S2D plots these contact probability plots. P1 interacts with extended and fully-collapsed TF very non-specifically with peaks distributed throughout its surface, though there are strong interaction sites at the ends. With semi-collapsed TF, P1 interacts more specifically and mostly through its supposed helices.This may be the result of much of Arm2 and available part of N-terminal being dominated by helices themselves.

In addition to the representative complex in the main text, figure S3A shows the starting and final conformations of the folded P1's complex with extended TF. They formed the touching complex, with most of the interaction sites lying at the ends of TF's appendages. The interactions are shown in the contact probability map of extended TF's residues (top panel), plotted in figure S3B. In addition, figure S3B also plots the contact probability maps of semi- and fully-collapsed TF residues (middle and bottom lower panels, respectively). In all cases, there are strong peaks on N-terminal, while those on the PPIase domain and the two arms (in extended TF) are absent in the case of the collapsed conformations of TF.

We performed Rosetta docking calculations on the folded P1 with extended TF (see Methods). Figure S4A shows three lowest energy conformations obtained from the calculations. Thereafter, we performed 100 ns long MD simulations on these starting structures, resulting in the formation of the complexes shown in figure S4B. TF collapsed around the P1 and encapsulated it to form, what we call, "hugging complexes" or HCs. In the process, the center of mass distance (COM) between TF and P1 decreased in the in HC1 and 3 (fig. S4C). However, the COM distance between TF and P1 remained nearly unchanged

in case of HC2, where it started at the lowest value among the three complexes. Regardless, in all cases, the number of contacts between the heavy atoms of TF and P1 increased – more so in case of HC3, likely strengthening the interactions. HC3 started with a similar configuration as HC1, with P1 bound between Arm1 and N-terminal. The substrate then lost contact with N-terminal, flipped over, and was trapped between PPIase domain and Arm1, leading to a conformation similar to HC2 (with a comparable number of contacts). It may indicate that the HC2 is indeed the most stable conformation, with strong interaction sites at Arm1 and inner surface of PPIase domain.

HCs1 and 2 were selected for Steered MD simulations to compare the unfolding landscapes of P1 in complex with TF and in isolation. The representative starting and final conformations of HCs 1 and 2, during these pulling experiments, are shown in figures S5A and B. The results of these simulations are presented in figure 3 in the main text. Here, we intend to show that the energy barriers in the work-extension graphs were not caused by the loss of native contacts of P1. Figures S5C plots the drop in the fraction of intact native C-α contacts of P1, in all three systems, during the pulling experiment. The average work-extension graphs are also plotted along the same x-axis of pulling distance, and show that the energy barriers in pulling in fact preceded the sudden drops in the native contacts. This may indicate that the energy barriers likely coincided with the breaking of PF-contacts, and that surpassing the barrier resulted in a quick unraveling of the protein's secondary structure. Figures S5D shows this phenomenon over a shorter pulling distance of 0.7 nm (first energy barrier), during which the PF-contacts are broken in all three systems. The precedence of surpassing of energy barrier to drop in the native contact fraction was less apparent in this regime than in the second energy barrier.

Figures S7A and S6A show the starting and final conformations of the extended TF's complexes with MBP and P2, respectively. Figures S7B shows the contact probability map of the interactions of the MBP's residues with extended TF. The peaks are discrete, suggesting that the interactions were specific. However, a closer look revealed that the interaction sites on MBP were dependent on the starting orientation of MBP with respect to TF. This was a likely result of MBP's size relative to the system, and the reduced size for P2 corrected this anomaly, as shown in figure S6B. P2's interaction sites are less specific and more uniformly distributed across its surface. This non-specificity in interactions allows P2 (and on a similar note, P1) to find more stable binding sites than MBP, which is one of the central themes of this paper.
