# Supplementary figures and images for: The Trigger Factor Chaperone Encapsulates and Stabilizes Partial Folds of Substrate Proteins
Source: PLoS Comput Biol. 2015 Oct 29;11(10):e1004444. doi: 10.1371/journal.pcbi.1004444 (PMC4626277; doi:10.1371/journal.pcbi.1004444)

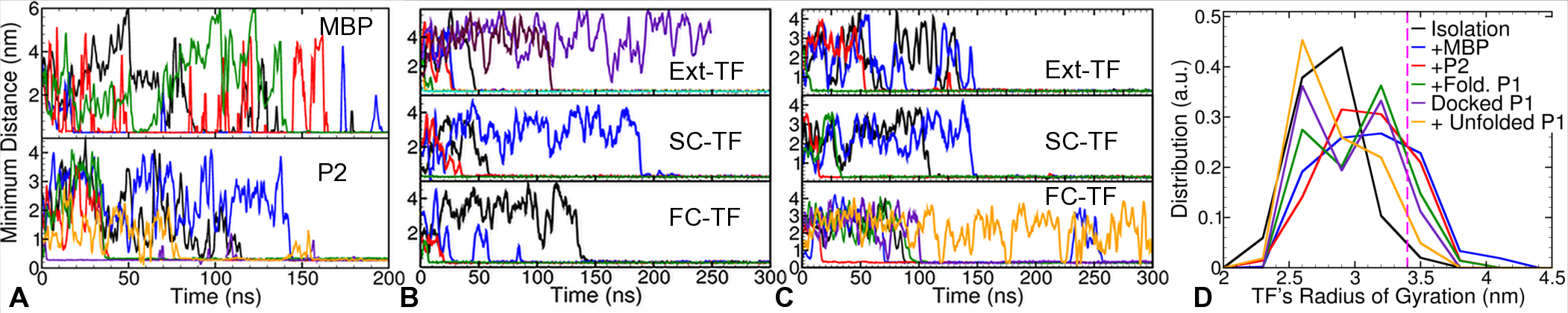

Supplement: S1 Fig — Minimum distance between the heavy atoms of TF and: A. MBP (top panel) and P2 (bottom panel); B. extended, semi-collapsed and fully-collapsed conformations of TF with Unfolded P1; C. extended, semi-collapsed and fully-collapsed conformations of TF with Folded P1; D. Distributions of final radii of gyration of TF in complex with different substrates. Black graph plots the same for TF in isolation. (TIF) [file pcbi.1004444.s003.tif]

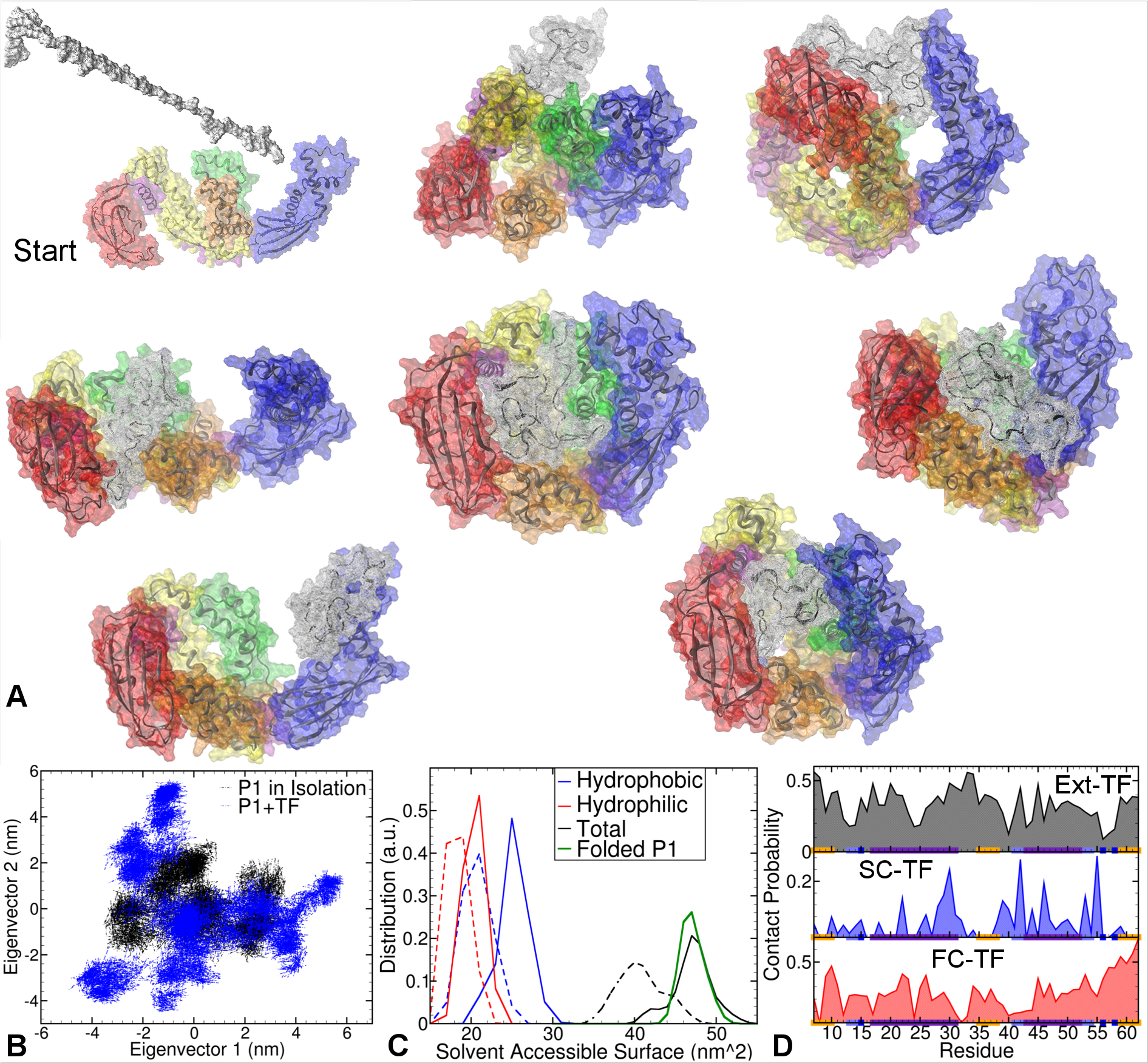

Supplement: S2 Fig — A. Starting and final (7) configurations of extended TF–Unfolded P1 simulations. P1 is shown in transparent white colored wire-mesh. B. 2-D projection along first two eigenvectors from dPCA of the combined dynamics of unfolded P1 in presence (blue) and absence (black) of TF. Unfolded P1 explores a larger conformational space in complex with extended TF than in isolation. C. Changes in the distribution of solvent accessible surface (SAS) area of unfolded P1 from the first 10 ns to the last 10 ns in presence of extended TF. Green graph plots the SAS area of folded P1 in absence of TF. D. Contact probabilities of the Unfolded P1 residues with extended, semi-collapsed and fully-collapsed conformations of TF. Barcode on the x-axis shows the supposed secondary structure of folded P1: orange regions for β–strands, indigo for α–helices, iceblue for turns, dark blue for bends, and the rest represents coil. (TIF) [file pcbi.1004444.s004.tif]

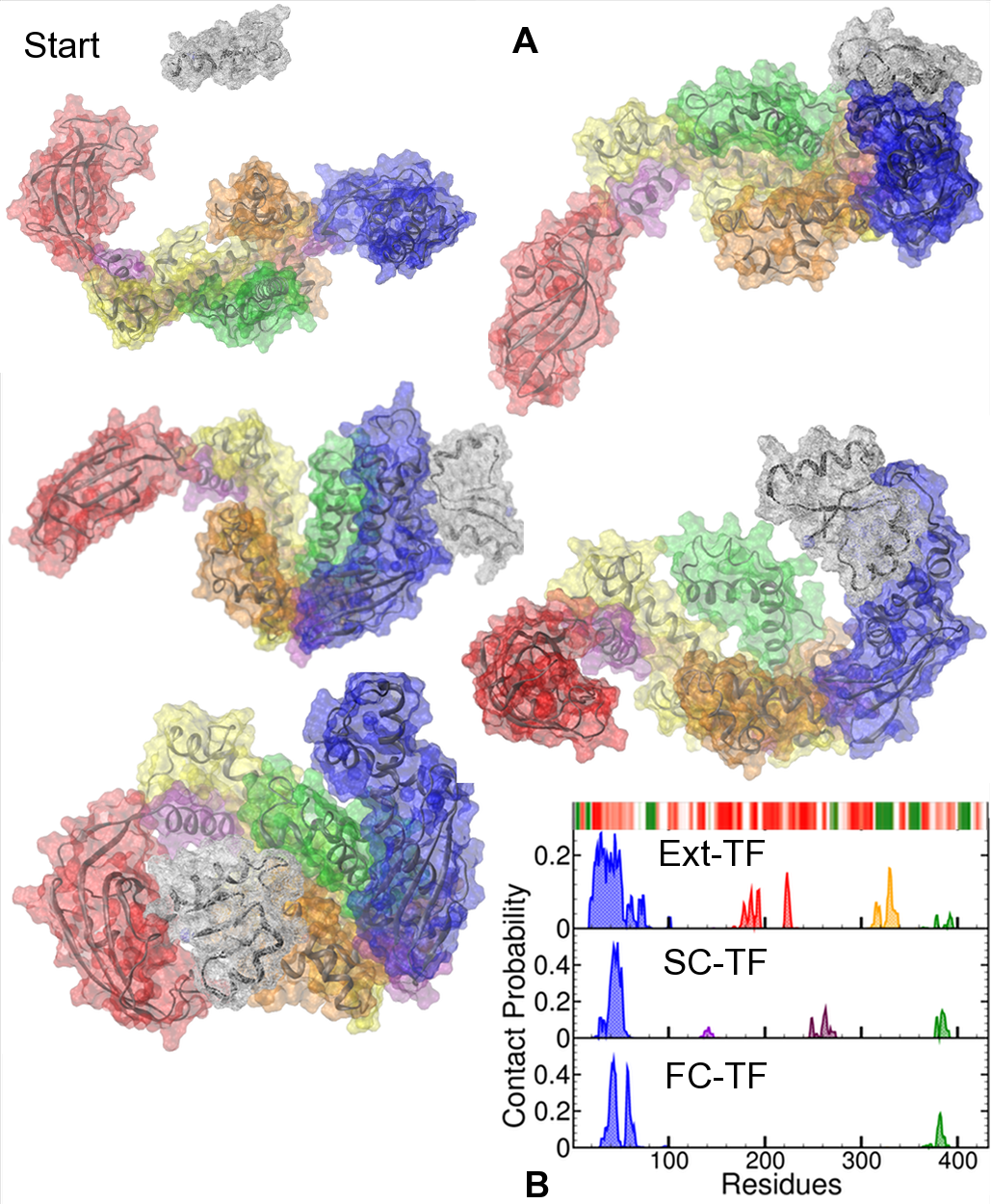

Supplement: S3 Fig — A. Starting and final (4) configurations of extended TF–Folded P1 simulations, forming “Touching Complexes.” P1 is shown in transparent white colored wire-mesh. B. Contact probabilities of the TF residues with Folded P1 in extended, semi-collapsed and fully-collapsed conformations of TF. (TIF) [file pcbi.1004444.s005.tif]

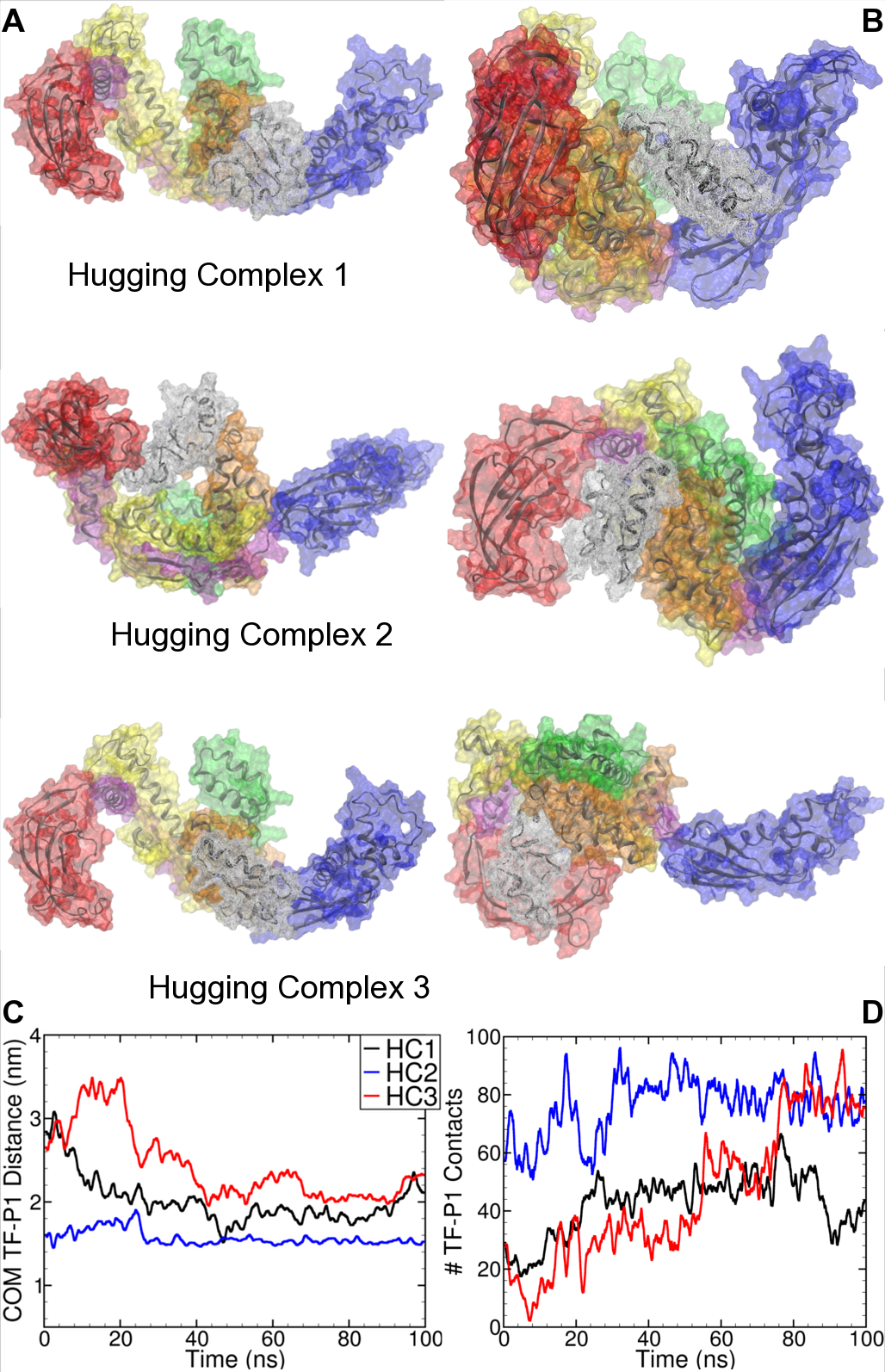

Supplement: S4 Fig — A. Starting configurations of “Hugging Complexes” (folded P1 docked on extended TF) as obtained from Rosetta docking calculations. B. Final configurations of the complexes after 100 ns of MD simulations. P1 is shown in transparent white colored wire-mesh. Time evolution of the C. centre of mass distance and D. number of contacts between TF and folded P1 over 100 ns. (TIF) [file pcbi.1004444.s006.tif]

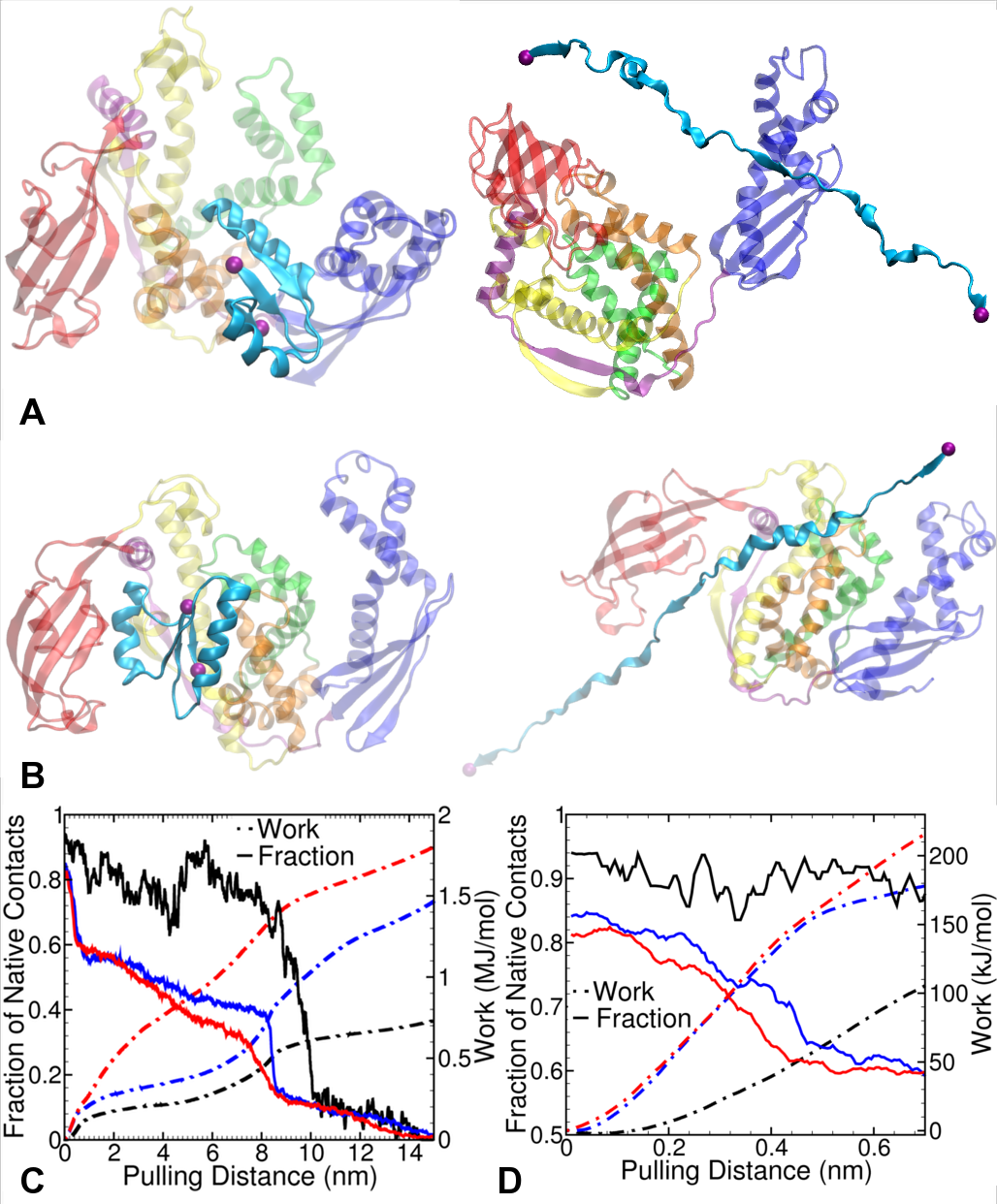

Supplement: S5 Fig — Representative structure of: A. System1 and B. System2, before and after pulling. Purple beads show the points at which the opposing pulling forces are applied. Change in the fraction of native C-α contacts of P1 over C. 15 nm of pulling that leads to unfolding. D. 1st 0.7 nm of pulling that leads of breaking of PF-contacts. Also plotted on the same time scale are the average work-extension graphs. Black graphs represent P1 in isolation, blue graphs show P1 in System 1, and red graphs show P1 in System 2. (TIF) [file pcbi.1004444.s007.tif]

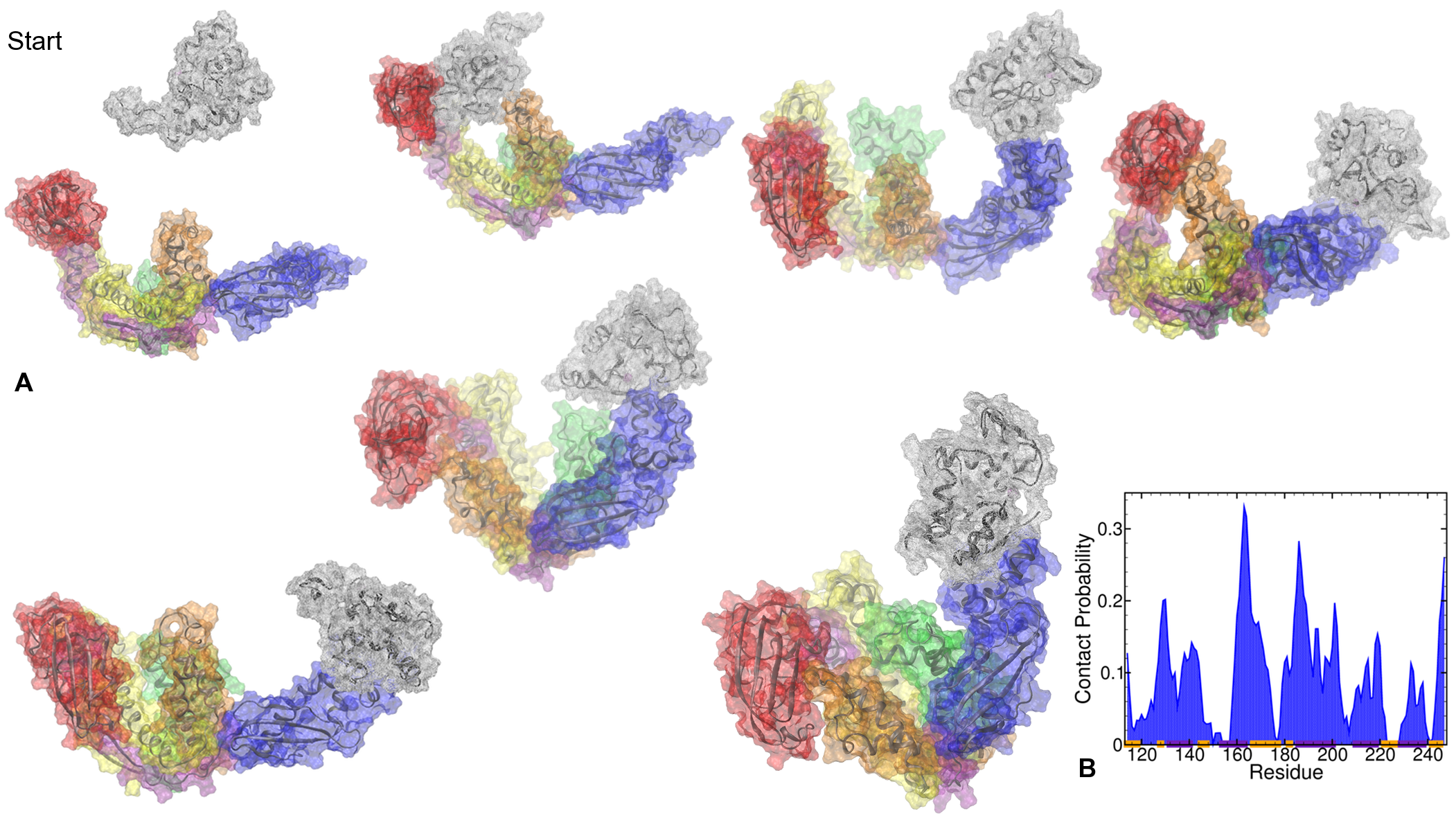

Supplement: S6 Fig — A. Starting and final (6) configurations of extended TF–P2 simulations. P2 is shown in transparent white colored wire-mesh. B. Contact probability of the P2 residues with extended conformation of TF. Barcode on the x-axis shows the supposed secondary structure of P2: orange regions for β–strands, indigo for α–helices, and the rest represents coil, bends and turns. (TIF) [file pcbi.1004444.s008.tif]

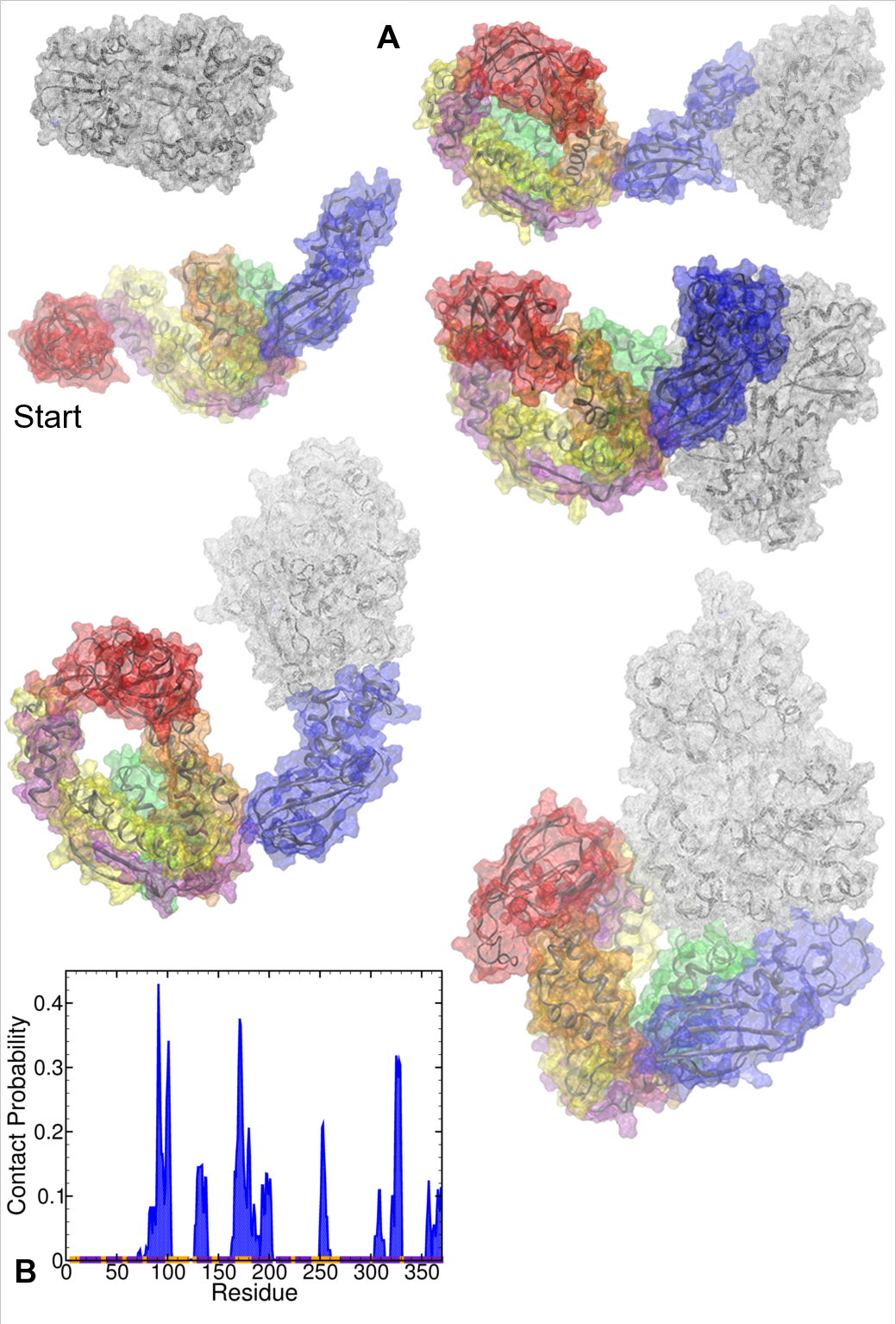

Supplement: S7 Fig — A. Starting and final (4) configurations of extended TF–MBP simulations. MBP is shown in transparent white colored wire-mesh. B. Contact probability of the MBP residues with extended conformation of TF. Barcode on the x-axis shows the supposed secondary structure of MBP: orange regions for β–strands, indigo for α–helices, and the rest represents coil, bends and turns. (TIF) [file pcbi.1004444.s009.tif]
